# Crucial role of estrogen for the mammalian female in regulating semen coagulation and liquefaction *in vivo*

**DOI:** 10.1371/journal.pgen.1006743

**Published:** 2017-04-17

**Authors:** Shuai Li, Marleny Garcia, Rachel L. Gewiss, Wipawee Winuthayanon

**Affiliations:** School of Molecular Biosciences, College of Veterinary Medicine, Washington State University, Pullman, Washington, United States of America; Cornell University, UNITED STATES

## Abstract

Semen liquefaction changes semen from a gel-like to watery consistency and is required for sperm to gain mobility and swim to the fertilization site in the Fallopian tubes. Kallikrein-related peptidases 3 (KLK3) and other kallikrein-related peptidases from male prostate glands are responsible for semen liquefaction by cleaving gel-forming proteins (semenogelin and collagen). In a physiological context, the liquefaction process occurs within the female reproductive tract. How seminal proteins interact with the female reproductive environment is still largely unexplored. We previously reported that conditional genetic ablation of *Esr1* (estrogen receptor α) in the epithelial cells of the female reproductive tract (*Wnt7a*^Cre/+^;*Esr1*^f/f^) causes female infertility, partly due to a drastic reduction in the number of motile sperm entering the oviduct. In this study, we found that post-ejaculated semen from fertile wild-type males was solidified and the sperm were entrapped in *Wnt7a*^Cre/+^;*Esr1*^f/f^ uteri, compared to the watery semen (liquefied) found in *Esr1*^f/f^ controls. In addition, semenogelin and collagen were not degraded in *Wnt7a*^Cre/+^;*Esr1*^f/f^ uteri. Amongst multiple gene families aberrantly expressed in the absence of epithelial ESR1, we have identified that a lack of *Klks* in the uterus is a potential cause for the liquefaction defect. Pharmacological inhibition of KLKs in the uterus replicated the phenotype observed in *Wnt7a*^Cre/+^;*Esr1*^f/f^ uteri, suggesting that loss of uterine and seminal KLK function causes this liquefaction defect. In human cervical cell culture, expression of several *KLKs* and their inhibitors (*SPINKs*) was regulated by estrogen in an ESR1-dependent manner. Our study demonstrates that estrogen/ESR1 signaling in the female reproductive tract plays an indispensable role in normal semen liquefaction, providing fundamental evidence that exposure of post-ejaculated semen to the suboptimal microenvironment in the female reproductive tract leads to faulty liquefaction and subsequently causes a fertility defect.

## Author summary

Semen liquefaction has been considered to be solely modulated by prostate-derived kallikrein-related peptidases (KLKs), especially KLK3 (or prostate specific antigen). However, our research demonstrated that female mice lacking estrogen receptor alpha (ERα) in the uterine epithelial cells had a drastic decrease in *Klk* transcripts and semen from fertile males fails to liquefy within the uteri of these females. Therefore, our results provide a novel aspect that, due to an interplay between semen and female reproductive tract secretions, the physiology of semen liquefaction is more complicated than previously assumed. This information will advance research on semen liquefaction in the female reproductive tract, an area that has never been explored, and could lead to the development of diagnostic tools for unexplained infertility cases and non-invasive contraception technologies.

## Introduction

In the United States, approximately 46% of women are unable to conceive within the first 12 months of trying to get pregnant [1]. Infertile couples may experience psychological distresses, including low self-esteem, isolation, and depression. Cumulatively, the infertile couples in the United States have spent more than ~$5 billion per year for diagnosis and treatment in fertility clinics [2]. These circumstances emphasize the need for a better understanding of the causes of infertility.

In humans, a semen coagulum is composed of the secretory products from male accessory organs, including the prostate glands, seminal vesicles, and coagulating glands. After ejaculation, both semen and sperm are deposited to the anterior wall of the vagina, adjacent to the ectocervical tissues. In order for the sperm to travel through the reproductive tract to fertilize the eggs in the oviduct (or Fallopian tube in humans) [3], the semen must undergo the process of liquefaction. Congenital absence, obstruction, or surgical removal of the seminal vesicles causes sterility in men and rodents [4, 5], indicating that not only are the secretory products from the seminal vesicles and prostate crucial for sperm motility, sperm viability, and chromatin stability of the sperm [6], but that they are also important for semen liquefaction.

Tissue kallikrein-related peptidases, or KLKs, are members of a serine protease family that exhibit trypsin- and chymotrypsin-like activities. Of the 37 *Klk* genes in the mouse genome, 26 encode functional proteins [7]. KLKs are translated as pre-pro-KLKs and are regulated by a proteolytic activation cascade that produces active KLKs, which are secreted from the kidneys, liver, salivary glands, and male and female reproductive organs [8, 9]. Sperm in the ejaculate are entrapped in a seminal coagulum, which is comprised mainly of semenogelins (SEMGs), fibronectin, and collagen secreted from the seminal vesicles [10, 11]. Liquefaction is mainly modulated by prostate derived KLK3 [10]. In females, KLKs 5–8, 10–11, and 13–15 are expressed at very high levels in the cervix and vagina compared to in other adult tissues [12, 13]. Moreover, KLK1 and KLK3 transcripts are expressed at the highest level in human endometrium when circulating estradiol (E_2_) is elevated [14, 15]. In rodents, E_2_ increases *KLK* expression in the uterus [16, 17]. These findings suggest that KLKs are expressed in the human and mouse reproductive tracts and that some of the KLKs in the uteri are regulated by E_2_. However, the role of the female reproductive tract in regulation of post-ejaculated seminal KLKs remains unclear.

E_2_ is a steroid hormone secreted from the granulosa cells of the ovary. Estrogens exert their functions through estrogen receptor α and β (ESR1 and ESR2). ESR1 is predominantly expressed in the female reproductive tissues, which include the ovary, oviduct, uterus, and mammary gland [18]. We previously reported that mice lacking ESR1 in the epithelial cells (using *Esr1*^f/f^ crossed with *Wnt7a*^Cre/+^ mice) are infertile [19], partly due to a reduction in the number of sperm able to reach the oviduct [20]. However, the effect of ESR1 loss in the epithelial cells on sperm transport in the uterus has not yet been investigated.

In this study, we showed 1) the expression of KLKs in the mouse uterus is downstream of E_2_ signaling acting through epithelial ESR1, and 2) loss of epithelial ESR1 disrupts sperm transport by affecting semen liquefaction and sperm motility. Additionally, as the semen is deposited at the ectocervix in humans, we examined whether human ectocervical cells express *KLK* transcripts and whether this expression is modulated by E_2_. Our studies provide the first evidence of how the interplay between semen and the female reproductive tract could impact fertility.

## Results

### Loss of ESR1 in uterine epithelial cells leads to a semen liquefaction defect in female mice

**Fig 1:**
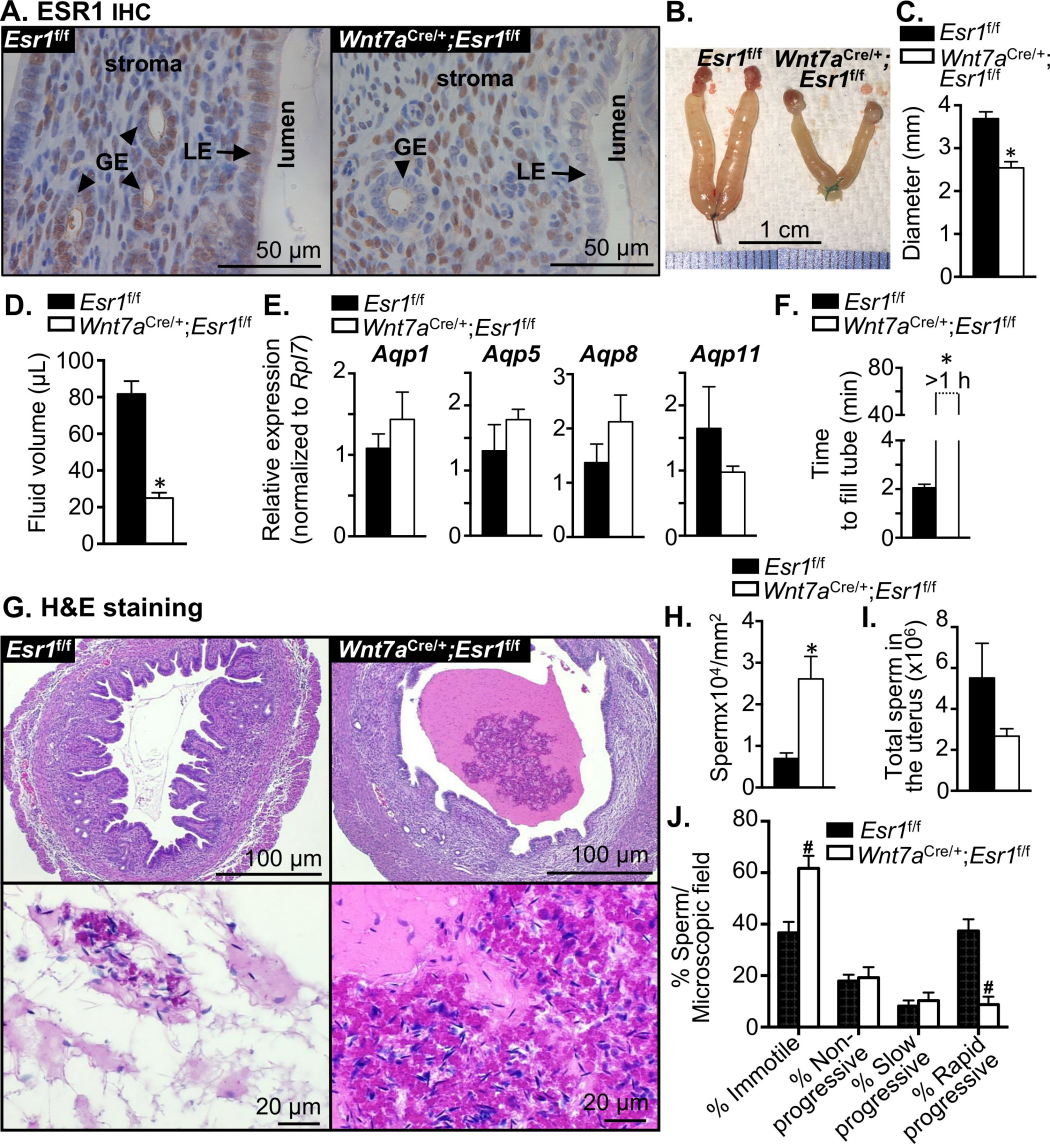
Loss of ESR1 in uterine epithelial cells leads to a semen liquefaction defect in female mice. **A.** Immunohistochemical analysis indicates the expression of ESR1 in the uteri of *Esr1*^f/f^ mice and its absence in *Wnt7a*^Cre/+^;*Esr1*^f/f^ animals at 0.5 dpc. LE = luminal epithelial cells; GE = glandular epithelial cells. **B.** Gross morphology of the reproductive tract of *Esr1*^f/f^ and *Wnt7a*^Cre/+^;*Esr1*^f/f^ animals at 0.5 dpc. **C.** Diameters of the uteri prior to dissection at 0.5 dpc, n = 9–10 mice/genotype. **D.** Total fluid volume (μL) collected from the uteri of *Esr1*^f/f^ and *Wnt7a*^Cre/+^;*Esr1*^f/f^ animals at 0.5 dpc, n = 3 mice/genotype. **E.** Expression of *Aqp* transcripts in the uteri at 0.5 dpc, n = 4–5 mice/genotype. **F.** Liquefaction time measured as the time it took for semen collected from the uteri at 0.5 dpc to fill a 25-μL capillary tube (minutes). Note that the liquefaction time for semen collected from *Wnt7a*^Cre/+^;*Esr1*^f/f^ uteri was over 1 h (>60 min), n = 3 mice/genotype. **G.** Hematoxylin & Eosin (H&E) staining of uterine samples collected at 0.5 dpc, n = 9–10 mice/genotype. **H.** Sperm density was determined from the semen within the uterine lumen per microscopic field of H&E stained images and normalized to mm^2^, n = 7–10 mice/genotype. **I.** Total sperm number in the uterus was calculated after normalization to the total fluid volume collected, n = 3 mice/genotype. **J.** Percentage of sperm motility per microscopic field in the semen collected from the uteri of *Esr1*^f/f^ and *Wnt7a*^Cre/+^;*Esr1*^f/f^ animals at 0.5 dpc, n = 3 mice/genotype. *, ^#^*p*<0.05; significant difference between *Esr1*^f/f^ and *Wnt7a*^Cre/+^;*Esr1*^f/f^ animals, unpaired *t-*test or two-way ANOVA, respectively. Representative images are shown. Graphs represent mean±SEM.

Our previous findings demonstrated that loss of ESR1 in the mouse uterine and oviductal epithelial cells causes a reduction of the number of sperm in the oviduct [20], however, the cause of this sperm reduction is unknown. Therefore, we evaluated the uterine morphology of these mice to gain insight of the potential explanation. To assess the effect of the absence of ESR1 in the uterine epithelial cells on sperm transport, the *Esr1*^f/f^ and *Wnt7a*^Cre/+^;*Esr1*^f/f^ females were mated with the WT male proven breeder and the uteri were collected at 0.5 dpc. The absence of ESR1 protein in the uterine luminal (LE) and glandular (GE) epithelial cells was confirmed in the *Wnt7a*^Cre/+^;*Esr1*^f/f^ compared to *Esr1*^f/f^ females using immunohistochemical (IHC) analysis (Fig 1A). Gross morphology of *Esr1*^f/f^ uteri collected at approximately 8 h after mating showed ballooning uteri, however, *Wnt7a*^Cre/+^;*Esr1*^f/f^ uteri did not (Fig 1B). As a result, the uterine diameter of *Wnt7a*^Cre/+^;*Esr1*^f/f^ animals was significantly smaller than those of *Esr1*^f/f^ controls (Fig 1C). In addition, total fluid volume from the uterine lumen was significantly lower in *Wnt7a*^Cre/+^;*Esr1*^f/f^ than in *Esr1*^f/f^ animals (Fig 1D).

Astwood demonstrated that E_2_ increases fluid accumulation in the uterine lumen and this E_2_-induced water imbibition is regulated through aquaporin (AQP) water channels [21, 22]. Our microarray analysis showed that E_2_ treatment robustly increased the expression of *Aqp1*, *Aqp5*, and *Aqp8* transcripts at 2 h, however, at 24 h, the induction of *Aqp* transcripts was not as strong as the 2 h treatment in the control uteri (S1 Table). These *Aqp* genes were expressed at significantly lower levels in *Wnt7a*^Cre/+^;*Esr1*^f/f^ compared to *Esr1*^f/f^ uteri 2 h or 24 h after E_2_ treatment, except *Aqp5* at 24 h (S1 Table). To determine whether *Aqps* were aberrantly expressed in *Wnt7a*^Cre/+^;*Esr1*^f/f^ at 8 h after mating, we collected uterine samples and performed real time RT-PCR analysis and found that the expression of the highly expressed *Aqps* in the uterus, including *Aqp1*, *Aqp5*, *Aqp8*, and *Aqp11*, were detected at comparable levels between *Esr1*^f/f^ and *Wnt7a*^Cre/+^;*Esr1*^f/f^ uteri at 0.5 dpc (Fig 1E). These findings suggest the water imbibition was rapidly mediated by E_2_ through an up-regulation of *Aqps* in control uteri. However, at 8 h after mating, transcript levels of *Aqps* were not differentially expressed in *Esr1*^f/f^ and *Wnt7a*^Cre/+^;*Esr1*^f/f^ uteri, regardless of a lack of ballooning in *Wnt7a*^Cre/+^;*Esr1*^f/f^ uteri.

Strikingly, semen liquefaction is affected by a lack of uterine epithelial ESR1. The liquefaction time was determined by the time it took for semen collected from *Esr1*^f/f^ and *Wnt7a*^Cre/+^;*Esr1*^f/f^ uteri to fill a 25-μL capillary tube. In *Esr1*^f/f^ uteri, the semen liquefaction time was 1.86±0.15 min, whereas the semen collected from *Wnt7a*^Cre/+^;*Esr1*^f/f^ uteri was too viscous and could not fill the tube by the end of the 1 h experimental time (Fig 1F). This evidence is the first illustration that semen from a WT male can be solidified as a result of exposure to the *Wnt7a*^Cre/+^;*Esr1*^f/f^ female reproductive tract, which could likely lead to the severely reduced sperm number in the oviduct observed in our previous findings [20].

Histological analysis of uterine cross-sections demonstrated that the semen present in the *Esr1*^f/f^ uterine lumen appears loose, whereas dense materials were observed only in the *Wnt7a*^Cre/+^;*Esr1*^f/f^ uteri (Fig 1G). The sperm density was measured to evaluate if the viscous semen or the dense materials within the *Wnt7a*^Cre/+^;*Esr1*^f/f^ uterine lumen contribute to the sperm entrapment/blockade within the uterus. We found the density of sperm/mm^2^ in the *Wnt7a*^Cre/+^;*Esr1*^f/f^ uteri was significantly higher in comparison to the sperm present in the *Esr1*^f/f^ uteri (Fig 1H). Because the sperm density was higher in the *Wnt7a*^Cre/+^;*Esr1*^f/f^ uteri, we assessed whether the total sperm count per seminal volume was different in *Esr1*^f/f^ and *Wnt7a*^Cre/+^;*Esr1*^f/f^ uteri. We found there was no significant difference in total sperm count between *Wnt7a*^Cre/+^;*Esr1*^f/f^ and *Esr1*^f/f^ animals 8 h after mating (Fig 1I). To determine whether the sperm present in the uteri were motile, we counted the percentage of motile sperm per microscopic field and found that the sperm collected from *Wnt7a*^Cre/+^;*Esr1*^f/f^ uteri had significantly higher numbers of immotile sperm compared to those from *Esr1*^f/f^ uteri (Fig 1J; S1 and S2 Videos). Conversely, a significantly smaller percentage of sperm with rapid progressive motility was observed in the semen collected from *Wnt7a*^Cre/+^;*Esr1*^f/f^ compared to those from *Esr1*^f/f^ uteri. This finding suggests that in the absence of uterine epithelial ESR1, normal sperm were unable to dislodge from the seminal coagulum, leading to a decreased number of motile sperm.

### Semen coagulants in the uterus in the absence of epithelial ESR1 are not degraded

**Fig 2:**
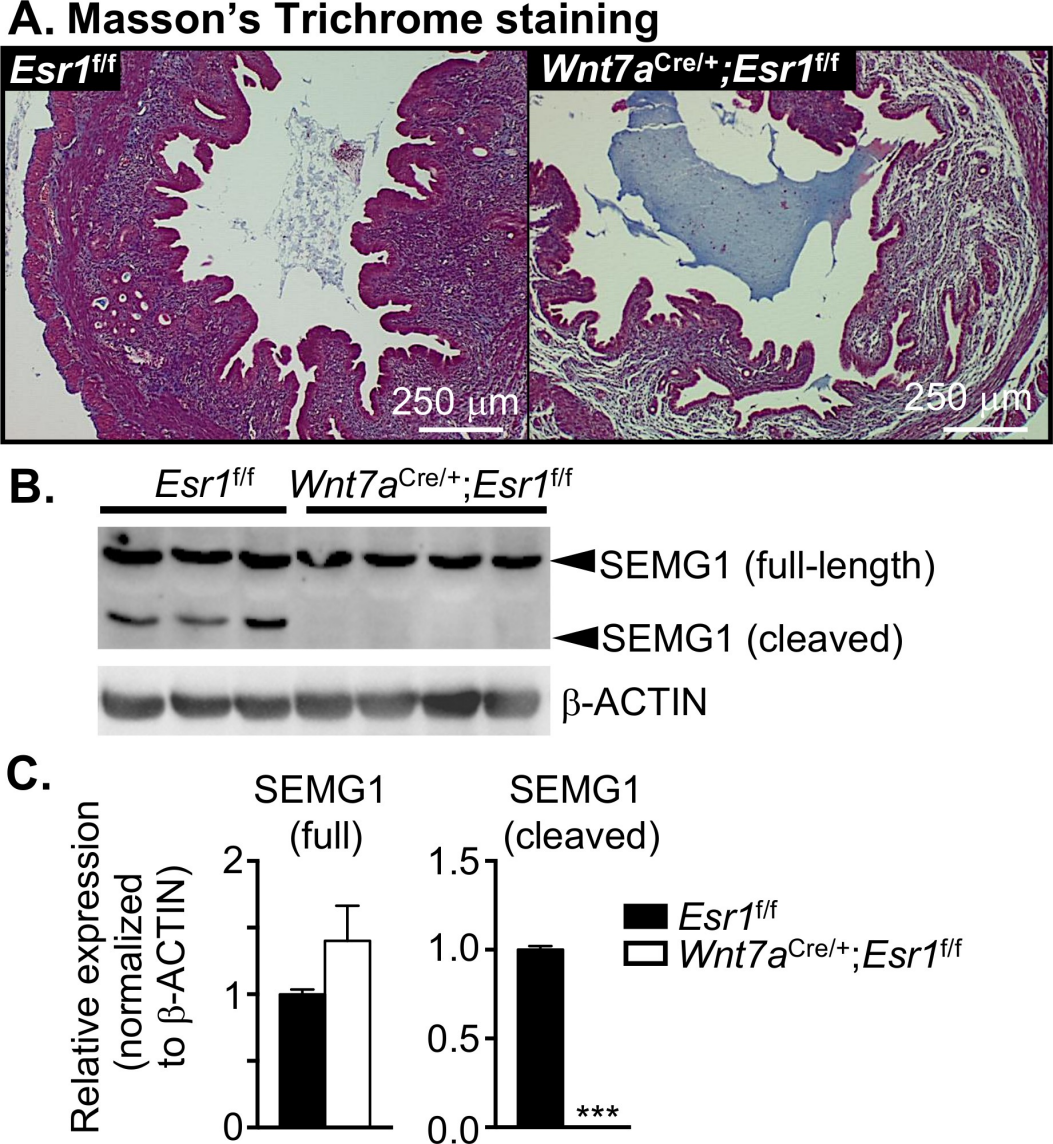
Gel-forming proteins are not degraded in *Wnt7a*^Cre/+^;*Esr1*^f/f^ uteri. **A.** Masson’s Trichrome staining of uterine cross-sections of *Esr1*^f/f^ and *Wnt7a*^Cre/+^;*Esr1*^f/f^ collected at 0.5 dpc. Blue staining represents collagen or connective tissues; red staining represents cytoplasm and muscle fibers. Representative images are shown. **B.** Immunoblotting of semenogelin 1 (SEMG1) in the uteri of *Esr1*^f/f^ and *Wnt7a*^Cre/+^;*Esr1*^f/f^ at 0.5 dpc. Upper bands indicate size of full length SEMG1 (~52kDa). Lower bands indicate the product size of cleaved SEMG1. β-Actin was used as a loading control (n = 3–4 mice/genotype). **C.** Quantitative analysis of SEMG1 protein expression normalized to β-Actin. ***p<0.001; significant difference between *Esr1*^f/f^ and *Wnt7a*^Cre/+^;*Esr1*^f/f^ animals, unpaired *t-*test. Graphs represent mean±SEM.

Degradation of semen coagulants such as collagen and SEMG1 leads to liquefaction [10, 11]. To determine whether the liquefaction defect observed in *Wnt7a*^Cre/+^;*Esr1*^f/f^ uteri was due to defective degradation of semen coagulants, we evaluated the presence of collagen and SEMG1 within the uterus after mating. Using Masson’s Trichrome staining, the results indicated that material present in the uterine lumen of *Wnt7a*^Cre/+^;*Esr1*^f/f^ animals contained a considerable amount of collagen compared to *Esr1*^f/f^ animals (Fig 2A). Additionally, measurable levels of cleaved SEMG1 were present only in the *Esr1*^f/f^ animals, and were not detected in *Wnt7a*^Cre/+^;*Esr1*^f/f^ animals (Fig 2B and 2C). This suggests that lacking uterine epithelial ESR1 contributes to a liquefaction defect due to reduced semen coagulant degradation.

### Uterine *Klk* transcripts are regulated by E_2_ signaling through epithelial ESR1

**Fig 3:**
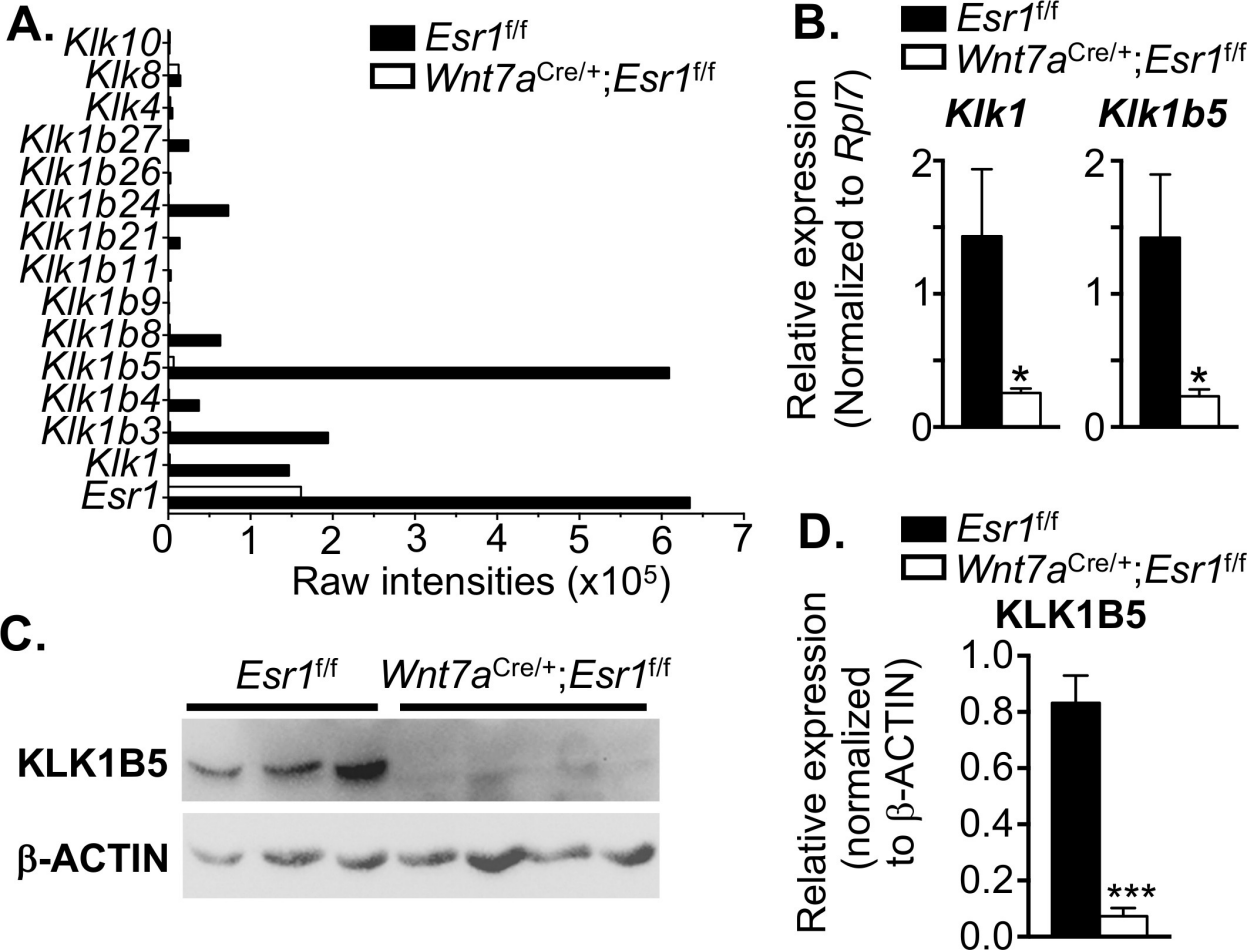
Uterine expression profile of *Klk* transcripts using microarray analysis. **A.** *Esr1*^f/f^ and *Wnt7a*^Cre/+^;*Esr1*^f/f^ females were ovariectomized and treated with E_2_ at 0.25 μg/mouse for 2 h. Expression values indicate raw intensities in E_2_ treated tissues in both *Esr1*^f/f^ and *Wnt7a*^Cre/+^;*Esr1*^f/f^ uteri (n = 3 mice/genotype/treatment). Expression level of *Esr1* was used as a reference to compare the overall gene expression level. **B.** Expressions of *Klk1* and *Klk1b5* were validated using real-time PCR from the uterine tissues collected at 0.5 dpc (n = 4–5 mice/genotype). **C.** Protein expression of KLK1B5 in the uteri of *Esr1*^f/f^ and *Wnt7a*^Cre/+^;*Esr1*^f/f^ animals at 0.5 dpc shown by immunoblotting. β-Actin was used as a loading control (n = 3–4 mice/genotype). **D.** Quantitative analysis of KLK1B5 protein expression normalized to β-Actin. *, ***p<0.05, 0.001; significant difference between *Esr1*^f/f^ and *Wnt7a*^Cre/+^;*Esr1*^f/f^ animals, unpaired *t-*test. Graphs represent mean±SEM.

In humans, it is known that KLK3 secreted from male accessory sex organs regulates semen liquefaction [10, 23]. However, we have reported here that liquefaction could also be modulated by the female reproductive tract. To elucidate whether the *Klk* family is present in the uterus and the expression is regulated by E_2_ signaling, our previously published microarray dataset of the uteri from *Esr1*^f/f^ and *Wnt7a*^Cre/+^;*Esr1*^f/f^ animals treated with E_2_ for 2 h [24] was analyzed. Transcript levels of the *Klk1* and *Klk1b* families were increased in E_2_ treated *Esr1*^f/f^ uteri at 2 h compared to vehicle treatment (Fig 3A). However, these E_2_-induced *Klk1* and *Klk1b* family expressions were minimally detected or were not at a detectable level in *Wnt7a*^Cre/+^;*Esr1*^f/f^ uteri compared to vehicle treatment. To validate expression at the transcriptional level in the presence of male ejaculates at 0.5 dpc, the mRNA expression of *Klk1* and *Klk1b5* was determined, as these two genes were abundant among other *Klk1* family members. *Klk1* and *Klk1b5* expression was significantly reduced in *Wnt7a*^Cre/+^;*Esr1*^f/f^ uteri compared to *Esr1*^f/f^ uteri (Fig 3B). Moreover, we found that the KLK1B5 protein level was significantly lower in the *Wnt7a*^Cre/+^;*Esr1*^f/f^ uteri compared to *Esr1*^f/f^ uteri at 0.5 dpc (Fig 3C and 3D).

### Decreased uterine *Klk* mRNA levels are not due to a lack of epithelial cell proliferation

**Fig 4:**
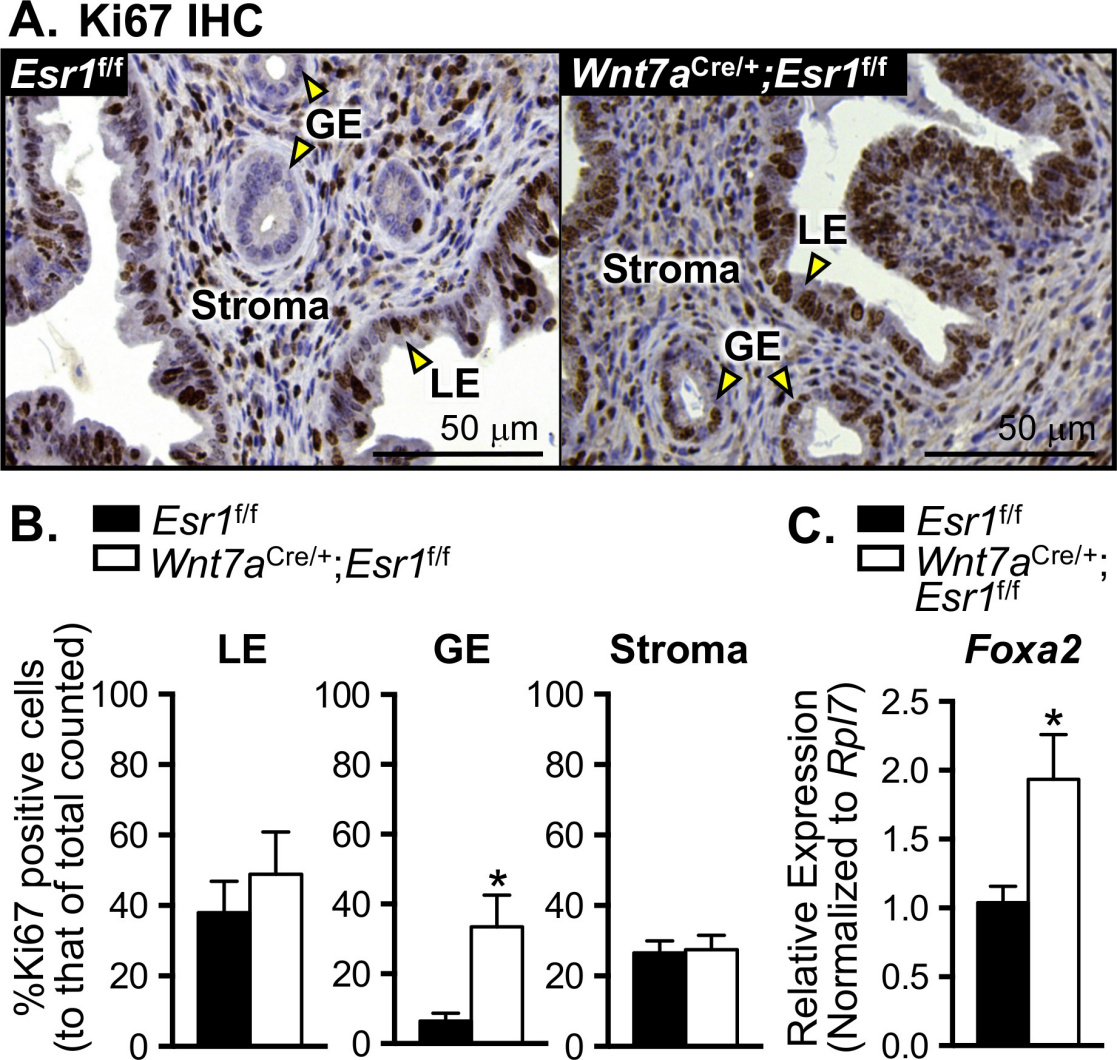
Luminal epithelial, glandular epithelial, and stromal cell proliferation in *Esr1*^f/f^ and *Wnt7a*^Cre/+^;*Esr1*^f/f^ animals at 0.5 dpc. **A.** Immunohistochemical staining (IHC) of Ki67 (proliferative marker) in the uterine cross-section from *Esr1*^f/f^ and *Wnt7a*^Cre/+^;*Esr1*^f/f^ animals. Representative images are shown. **B.** Percentage of Ki67 positive cells of total cell type counted within microscopic field (3 fields per animal). Total cell counts for luminal epithelium (LE), glandular epithelium (GE), and stroma are 3058, 939, and 9160 cells, respectively. **C.** Expression of *Foxa2* in whole uteri collected from *Esr1*^f/f^ and *Wnt7a*^Cre/+^;*Esr1*^f/f^ animals at 0.5 dpc determined by real-time RT-PCR analysis. *p<0.05; significant difference between *Esr1*^f/f^ and *Wnt7a*^Cre/+^;*Esr1*^f/f^ animals, unpaired *t-*test. n = 4–10 mice/genotype. Graphs represent mean±SEM.

It has been previously shown that *Klk1* mRNA is expressed in the endometrial glands of humans and mice [15, 17]. Therefore, glandular epithelial cells may be the potential source of KLK production in the uterine tissues. To determine whether the loss of uterine epithelial ESR1 affects cell proliferation and subsequently disrupts the *Klk* transcript levels, we evaluated the proliferation of all uterine cell types including luminal, glandular, and stromal cells at 0.5 dpc in *Esr1*^f/f^ and *Wnt7a*^Cre/+^;*Esr1*^f/f^ animals. Ki67 was used as a marker to visualize cell proliferation. The percentages of Ki67 positive luminal epithelial and stromal cells were comparable between *Esr1*^f/f^ and *Wnt7a*^Cre/+^;*Esr1*^f/f^ uteri (Fig 4A and 4B). However, Ki67 positive glandular epithelial cells were detected at a significantly higher level in *Wnt7a*^Cre/+^;*Esr1*^f/f^ than those of *Esr1*^f/f^ uteri (Fig 4B). Additionally, the marker of the gland, *Foxa2*, was expressed at a significantly higher level in *Wnt7a*^Cre/+^;*Esr1*^f/f^ compared to *Esr1*^f/f^ uteri (Fig 4C). These results suggest that a lack of *Klk* transcripts in the absence of epithelial ESR1 was not due to a loss of glandular epithelial cells; in fact, the marker of glandular epithelial cells was expressed at a higher level in uteri lacking epithelial ESR1.

### Lacking epithelial ESR1 leads to increased serine protease inhibitor transcripts, but not SERPINH1 protein

Human KLK activity can be effectively inhibited by endogenous protease inhibitors such as serine protease inhibitors (SERPINs), serine protease inhibitors, Kazal type (SPINKs), elafin, and α1-antitrypsin [25, 26]. It has been previously shown that SERPIN inhibits KLK activity by interacting with the reactive loop, causing an irreversible protein conformational change of KLK [27, 28]. In addition, changes in expression levels of other proteinases and protease inhibitors could also affect KLK activity. Therefore, to explore whether the liquefaction defect observed in *Wnt7a*^Cre/+^;*Esr1*^f/f^ uteri was due to aberrant expression of protease inhibitors, proteinases, and proteinase inhibitors, in addition to a loss of *Klk* production, the microarray dataset of E_2_ treated *Esr1*^f/f^ and *Wnt7a*^Cre/+^;*Esr1*^f/f^ uteri was assessed (Table 1). To compare the expression level of proteases and protease inhibitors, we listed the signal intensities of genes relative to *Esr1* in Table 1. *Esr2* was used as a negative control. We found that 7 of 22 *Serpin* gene family members had higher signal intensities, whereas the intensities of 3 of 22 *Serpins* were lower in *Wnt7a*^Cre/+^;*Esr1*^f/f^ compared to *Esr1*^f/f^ uteri (Table 1). However, 54% (12 of 22) of the *Serpin* genes in *Wnt7a*^Cre/+^;*Esr1*^f/f^ were expressed at comparable levels to those of *Esr1*^f/f^ uteri. In addition, we evaluated the expression of other proteinases and proteinase inhibitors such as *Spinks*, matrix metalloprotenases (*Mmps*), tissue inhibitor of metalloproteinases (*Timps*), cysteine proteases and protease inhibitors, which include defensins (*Def*), cathepsins (*Cts*), α2-macrogobulin (*A2m*), and secretory leukocyte peptidase inhibitor (*Slpi*). The expression levels of these proteases and protease inhibitors were at comparable levels between E_2_-treated *Wnt7a*^Cre/+^;*Esr1*^f/f^ and E_2_-treated *Esr1*^f/f^ uteri (Table 1). The only exceptions were the expression of *Def3* was significantly lower and *Ctsw* was significantly higher in E_2_-treated *Wnt7a*^Cre/+^;*Esr1*^f/f^ uteri compared to E_2_-treated *Esr1*^f/f^ uteri.

**Table 1:** Microarray analysis showing all the transcript levels^π^ of proteases, proteinases, and their inhibitors detected in the uterus of *Esr1*^f/f^ and *Wnt7a*^Cre/+^;*Esr1*^f/f^ 2 h after E_2_ treatment.

Accession #	Primary Sequence Name	*Esr1*^f/f^ E_2_ 2h		*Wnt7a*^Cre/+^;*Esr1*^f/f^ E_2_ 2h		Fold Change
		Signal intensity	*p*-value	Signal intensity	*p*-value	
**Estrogen Receptors (*Esr*)**∫						
NM_007956	*Esr1*	63,423.7	0.0E+00	16,143.2	3.3E-14	-3.9
NM_207707	*Esr2*	10.6	9.0E-05	14.8	2.0E-04	1.4
**Serine protease inhibitors (*Serpin*)**						
NM_009244	*Serpina1b*	190.4	1.1E-09	1,359.7	2.6E-24	7.1
NM_009246	*Serpina1d*	7,489.0	5.7E-07	34,335.3	3.0E-19	4.6
NM_009247	*Serpina1e*	167.7	7.9E-11	1,096.1	0.0E+00	6.5
M64085	*Serpina3g*	1,609.0	1.7E-16	2,174.6	0.0E+00	1.4
NM_009253	*Serpina3m*	449.2	2.2E-11	239.9	3.3E-12	-1.9
NM_009252	*Serpina3n*	272.4	5.3E-06	280.6	2.0E-05	1.0
NM_198028	Serpinb10-ps*	146.0	9.2E-11	1,338.9	2.1E-02	9.2
NM_025867	*Serpinb11*	8,716.5	5.6E-09	16,167.9	6.6E-03	1.9
NM_025429	*Serpinb1a*	7,391.3	1.1E-31	9,023.8	0.0E+00	1.2
NM_173051	*Serpinb1c*	911.1	1.0E-19	801.1	0.0E+00	-1.1
NM_009254	*Serpinb6a*	17,304.3	0.0E+00	15,181.6	0.0E+00	-1.1
NM_011454	*Serpinb6b*	5,768.6	0.0E+00	4,993.1	0.0E+00	-1.2
NM_148942	*Serpinb6c*	1,276.8	0.0E+00	790.3	2.2E-31	-1.6
NM_027548	*Serpinb7*	469.5	0.0E+00	5,919.6	6.7E-03	12.6
NM_011459	*Serpinb8*	223.3	1.3E-16	437.6	3.5E-12	2.0
NM_009256	*Serpinb9*	765.9	2.5E-14	825.8	8.8E-08	1.1
NM_008871	*Serpine1*	2,212.4	6.0E-29	1,959.2	4.6E-17	-1.1
AK046232	*Serpine2*	1,121.3	9.5E-12	935.3	1.7E-29	-1.2
NM_011340	*Serpinf1*	44,423.2	0.0E+00	40,009.5	0.0E+00	-1.1
NM_008878	*Serpinf2*	108.0	0.0E+00	102.9	1.2E-11	-1.0
NM_009776	*Serping1*	25,991.3	1.2E-28	33,053.9	0.0E+00	1.3
NM_009825	*Serpinh1*	228,658.0	0.0E+00	136,746.8	0.0E+00	-1.7
**Serine Protease Inhibitors, Kazal Type (*Spink*)**						
NM_183284	*Spink2*	66.0	9.3E-31	250.6	3.1E-09	3.8
NM_009258	*Spink3*	9,864.3	0.0E+00	8,934.6	0.0E+00	-1.1
NM_183136	*Spink8*	540.3	2.3E-03	515.4	1.2E-06	-1.0
NM_030061	*Spink12*	154.1	3.9E-07	139.6	1.0E-05	-1.1
**Matrix Metalloproteinases (*Mmp*)**						
NM_008610	*Mmp2*	22,720.9	0.0E+00	27,531.3	0.0E+00	1.2
NM_010809	*Mmp3*	2,191.8	2.4E-04	2,764.3	0.0E+00	1.3
NM_010810	*Mmp7*	146.1	1.4E-15	342.0	4.0E-14	2.3
NM_008611	*Mmp8*	35.4	1.4E-02	124.7	2.3E-02	3.5
NM_013599	*Mmp9*	584.6	2.6E-12	678.0	1.3E-25	1.2
NM_019471	*Mmp10*	846.8	3.8E-20	457.8	2.4E-06	-1.8
NM_008606	*Mmp11*	15,894.3	4.3E-13	26,041.9	1.0E-05	1.6
NM_008605	*Mmp12*	636.8	3.2E-03	896.6	1.0E-04	1.4
NM_008607	*Mmp13*	14.6	4.7E-03	10.5	1.7E-02	-1.4
NM_008608	*Mmp14*	55,741.3	6.4E-15	67,370.4	2.0E-22	1.2
NM_008609	*Mmp15*	740.4	3.6E-22	1,771.6	1.6E-17	2.4
AK034828	*Mmp16*	1,256.7	0.0E+00	1,149.2	0.0E+00	-1.1
NM_011846	*Mmp17*	18,790.8	1.9E-13	9,951.8	7.7E-08	-1.9
NM_021412	*Mmp19*	1,237.7	0.0E+00	976.1	4.8E-04	-1.3
NM_013903	*Mmp20*	3.6	5.8E-01	2.6	5.9E-01	-1.4
NM_152944	*Mmp21*	11.6	4.2E-08	8.5	3.3E-02	-1.4
NM_011985	*Mmp23*	3,754.6	0.0E+00	4,186.8	0.0E+00	1.1
NM_010808	*Mmp24*	113.1	1.7E-03	63.4	1.0E-11	-1.8
**Tissue Inhibitor of Metalloproteinases (*Timp*)**						
NM_011593	*Timp1*	2,404.3	0.0E+00	2,775.3	1.6E-08	1.2
NM_011594	*Timp2*	125,039.8	0.0E+00	175,180.4	7.6E-24	1.4
NM_011595	*Timp3*	132,754.1	0.0E+00	118,598.0	0.0E+00	-1.1
NM_080639	*Timp4*	254.3	2.4E-38	156.7	0.0E+00	-1.6
**Other Cysteine Proteases: Cathepsin (*Cts*) and Defensins (*Def*)**						
NM_007850	*Defa3*	12,116.57	5.9E-09	1,945.88	2.4E-39	-6.2
NM_145157	*Defb19*	116.50	2.2E-34	98.33	0.0E+00	-1.2
NM_008906	*Ctsa*	16,859.07	0.0E+00	23,719.12	0.0E+00	1.4
NM_007798	*Ctsb*	206,338.64	0.0E+00	140,848.03	0.0E+00	-1.2
NM_009982	*Ctsc*	6,017.71	2.9E-16	6,358.88	1.1E-17	1.1
NM_009983	*Ctsd*	72,181.23	0.0E+00	98,438.81	2.8E-45	1.4
NM_007799	*Ctse*	206.77	1.7E-18	248.40	1.2E-15	1.2
NM_019861	*Ctsf*	5,795.26	0.0E+00	8,056.69	9.6E-25	1.4
NM_007801	*Ctsh*	66,238.02	0.0E+00	58,639.07	0.0E+00	-1.2
NM_007802	*Ctsk*	2,400.70	9.3E-11	3,140.82	1.5E-28	1.3
NM_009984	*Ctsl*	143,215.02	0.0E+00	113,895.86	0.0E+00	-1.3
NM_177662	*Ctso*	8,020.15	1.2E-17	11,758.58	0.0E+00	1.5
NM_021281	*Ctss*	47,364.07	8.7E-19	63,009.99	7.7E-28	1.3
NM_009985	*Ctsw*	1,121.71	1.4E-24	2,135.26	0.0E+00	1.9
NM_022325	*Ctsz*	36,867.16	0.0E+00	27,974.15	1.0E-05	-1.3
**Other Protease Inhibitors:** α**2-macroglobulin (*A2m*) and Secretory Leukocyte Peptidase Inhibitor (*Spli*)**						
NM_175628	*A2m*	264.6	8.3E-09	304.7	5.3E-08	1.2
NM_011414	*Slpi*	6,455.48	1.3E-29	4,843.85	4.5E-42	-1.3

π All the transcripts detected in each gene family in the uteri were listed in the table regardless of the fold changes. Transcript levels indicated by the raw signal intensities; cut off values were ≤ 100 in both groups.

∫ *Esr1* expression level was used to compare to the expression to other genes, *Esr2* was used as a negative control, in which level of expression is less than the intensity of 100.

* pseudogene

**Fig 5:**
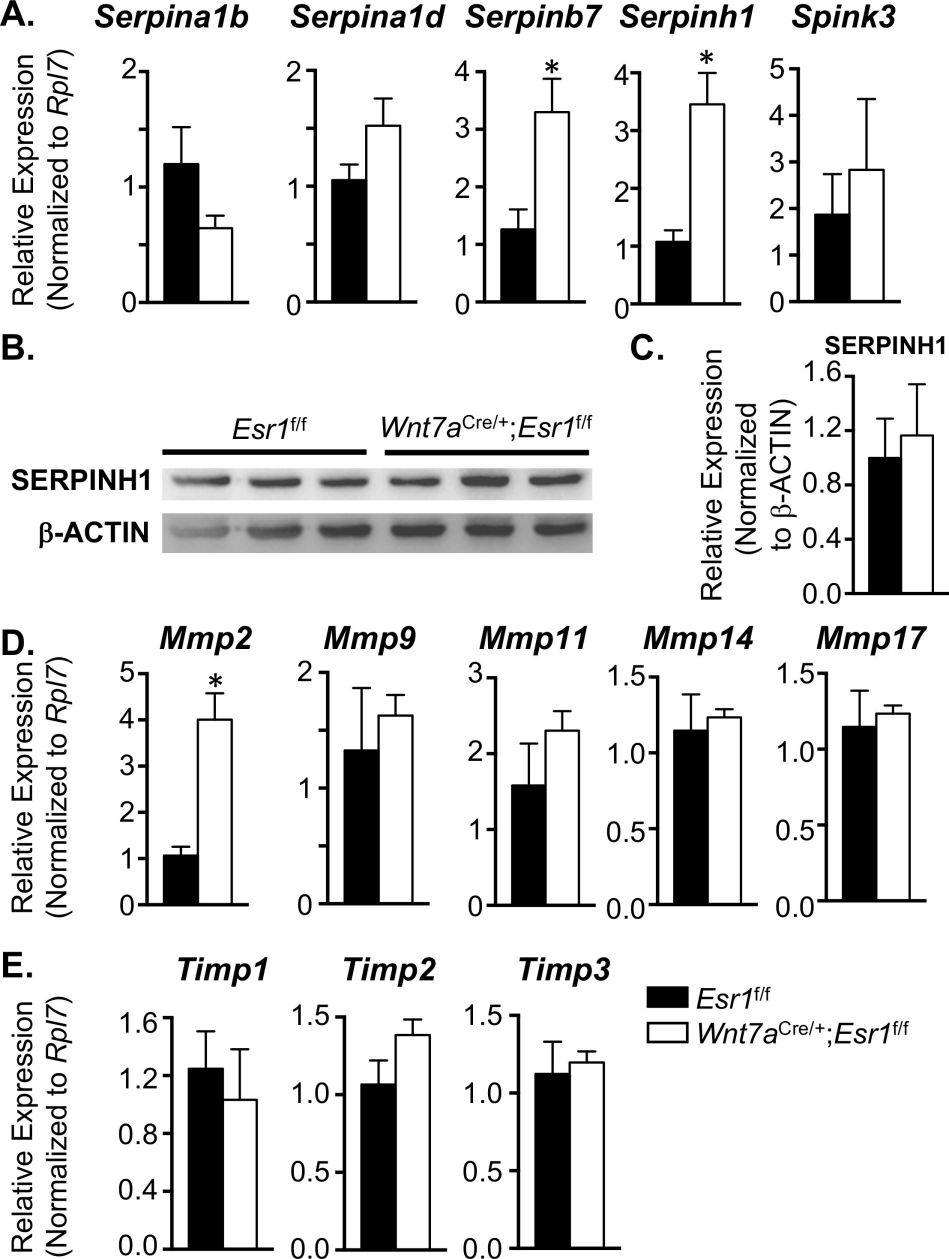
Expression of other proteases and protease inhibitors in the uterus. Transcript levels of **A.** protease inhibitors (*Serpina1b*, *Serpina1d*, *Serpinb7*, *Serpinh1*, and *Spink3*) in *Esr1*^f/f^ and *Wnt7a*^Cre/+^;*Esr1*^f/f^ uteri at 0.5 dpc. **B.** and **C.** Expression of SERPINH1 normalized to β-ACTIN levels in *Esr1*^f/f^ and *Wnt7a*^Cre/+^;*Esr1*^f/f^ uteri at 0.5 dpc (n = 3 mice/genotype). Transcript levels of **D.** matrix metalloproteinases *(Mmp2*, *Mmp9*, *Mmp11*, *Mmp14*, and *Mmp17*) and **E.** tissue inhibitor of metalloproteinases 1 (*Timp1*, *Timp2*, and *Timp3*) in *Esr1*^f/f^ and *Wnt7a*^Cre/+^;*Esr1*^f/f^ uteri at 0.5 dpc. Expression values indicate fold change over *Esr1*^f/f^ (n = 4–5 mice/genotype). *p<0.05; significant difference between *Esr1*^f/f^ and *Wnt7a*^Cre/+^;*Esr1*^f/f^ animals, unpaired *t-*test. Graphs represent mean±SEM.

To identify whether these genes were expressed and regulated by epithelial ESR1 during early pregnancy, we collected the uterine tissues at 0.5 dpc from *Esr1*^f/f^ and *Wnt7a*^Cre/+^;*Esr1*^f/f^ animals and evaluated the expression levels using real-time PCR analysis. *Serpina1b*, *Serpina1d*, *Serpinb7*, *Serpinh1*, *Spink3*, *Mmp2*, *Mmp9*, *Mmp11*, *Mmp14*, *Mmp17*, *Timp1*, *Timp2*, and *Timp3* genes were selected for real-time PCR analysis based on their moderate to high levels of signal intensities (from the microarray dataset mentioned in the previous section) compared to other genes in the families. We found that *Serpina1b*, *Serpina1d*, and *Spink3* were expressed at comparable level between *Esr1*^f/f^ and *Wnt7a*^Cre/+^;*Esr1*^f/f^ uteri at 0.5 dpc (Fig 5A). However, expression of *Serpinb7* and *Serpinh1* were significantly higher in *Wnt7a*^Cre/+^;*Esr1*^f/f^ compared to *Esr1*^f/f^ uteri (Fig 5A). We evaluated the protein expression of the most abundant uterine SERPIN, SERPINH1, using immunoblotting and found that although the transcription level of *Serpinh1* was significantly higher in *Wnt7a*^Cre/+^;*Esr1*^f/f^ compared to *Esr1*^f/f^ uteri, the translation of SERPINH1 was at comparable levels (Fig 5B and 5C). In addition, only *Mmp2*, not other *Mmps* nor *Timps*, was expressed at a significantly higher level in *Wnt7a*^Cre/+^;*Esr1*^f/f^ compared to *Esr1*^f/f^ uteri (Fig 5D and 5E). These results suggest that E_2_ not only regulates the expression of *Klk* transcripts, but that it also regulates the transcription of serine protease inhibitors (*Serpinb7 and Serpinh1*). However, SERPINH1 protein level was not altered in the absence of uterine epithelial ESR1.

**Fig 6:**
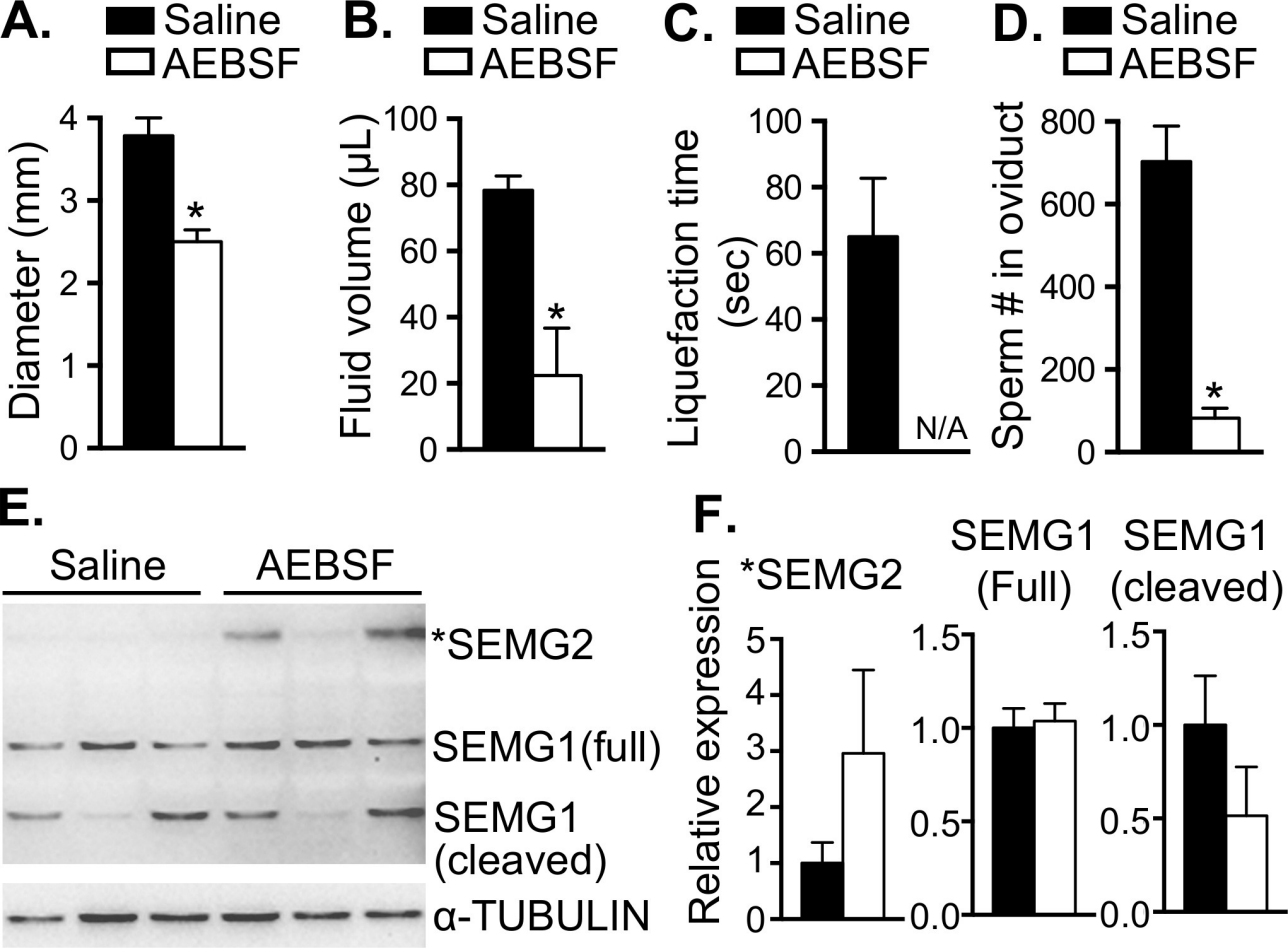
Treatment of serine protease inhibitor (AEBSF) in the uterus decreases the number of sperm in the oviduct. **A.** Uterine diameter (mm), **B.** Intraluminal fluid volume (μL), **C.** Liquefaction time (seconds) of the semen collected from the uteri, and **D.** Total number of sperm in the oviducts at 0.5 dpc after the transcervical injection of 15 μL saline or 300 μg of AEBSF. **E.** Immunoblotting of the uterine protein (10 μg/lane) collected from the uteri of mice treated with saline or AEBSF using SEMG1 antibody. *SEMG2 are the bands at the predicted size of SEMG2 protein. **F.** Quantitative analysis of signal intensities of *SEMG2, SEMG1 full-length, and SEMG1 cleaved normalized to α-TUBULIN protein. *p<0.05; significant difference between saline and AEBSF treatment, unpaired *t-*test. Graphs represent mean±SEM, n = 3–6 mice/group. N/A; not available as the amount of semen collected from the uteri of mice treated with AEBSF (majority of the mice had less than 20 μL of the semen volume) was insufficient to measure the liquefaction time.

To explore the possibility that alteration of KLK activity in the uteri can disrupt the post-ejaculated semen liquefaction process, we injected into the uterine lumen a tissue KLK inhibitor (4-(2-Aminoethyl)benzenesulfonyl fluoride hydrochloride or AEBSF) to inhibit serine proteases including multiple tissues’ KLKs [29]. Note that KLKs in the uterus of a mated female could originate from both the semen and the female reproductive tract. Compared to the *Wnt7a*^Cre/+^;*Esr1*^f/f^ uteri, which lack *Klk* transcripts, the AEBSF treatment stimulated a significant reduction in KLK activities without an alteration of other E_2_-targeted pathways. The uterine diameter as well as intrauterine fluid volume of semen collected from female mice treated with AEBSF were significantly reduced compared to those of saline treated mice (Fig 6A and 6B). However, we were unable to determine the liquefaction time of the semen collected from AEBSF treated animals, as the volume of semen was insufficient for measurement (Fig 6C). More importantly, the number of sperm that reached the oviducts was drastically decreased in the female mice treated with AEBSF compared to the saline treated controls (Fig 6D). In addition, we found that the SEMG2 protein tended to be expressed at a higher level in the AEBSF treated group, whereas the cleaved SEMG1 protein tended to be lower in the AEBSF compared to the saline treated group (Fig 6E and 6F). This finding indicates that the use of a pharmacological inhibitor of serine protease activity (including KLKs) recapitulates the phenotype observed in *Wnt7a*^Cre/+^;*Esr1*^f/f^ uteri.

### Human ectocervical and endocervical cells express *KLKs*

**Fig 7:**
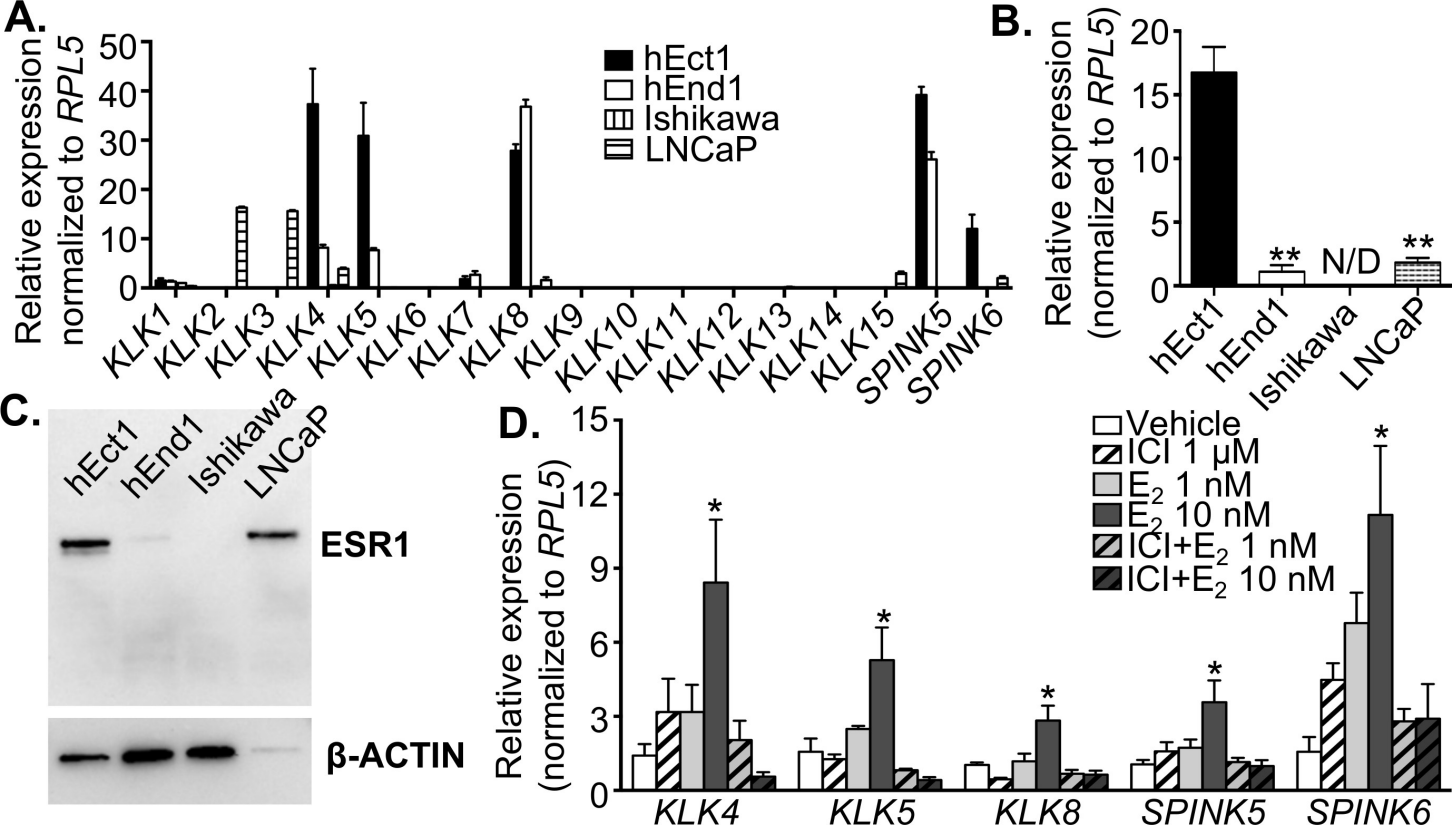
Endogenous gene expression of *KLK* and serine protease inhibitor (*SPINK*) in human cell lines. **A.** Expression levels of *KLK1-15*, *SPINK5* and *SPINK6* in immortalized human ectocervical (hEct1), endocervical (hEnd1), uterine adenocarcinoma (Ishikawa), and prostate cancer (LNCaP) cells in the absence of any hormonal treatment. Note that undetectable values were plotted as expression levels of 0 in the graph. **B.** Transcript levels of *ESR1* normalized to *RPL5* in hEct1, hEnd1, Ishikawa, and LNCaP cell lines. **p<0.01; significant difference when compared to hEct1 cells, one-way ANOVA analysis. N/D = not detectable. **C.** Immunoblotting analysis of ESR1 protein expression levels compared to the β-ACTIN loading control in hEct1, hEnd1, Ishikawa, and LNCaP cell lines. **D.** Expression of *KLK4*, *KLK5*, *KLK8*, *SPINK5* and *SPINK6* in hEct1 cells after 24 h treatment of vehicle (ethanol), ICI182,780 (ICI) at a dose of 1 μM, 17β-estradiol (E_2_) at doses of 1 or 10 nM, or a combination of ICI (1 μM) and E_2_. Expression values were relative to *RPL5*, mean±SEM. *p<0.05; significant difference when compared to vehicle, one-way ANOVA analysis.

Due to anatomical differences in the location of semen after ejaculation between mice and humans, as well as the difference between human and mouse KLKs, we tested whether human cervical cells, particularly ectocervical (hEct1) and endocervical (hEnd1) cells, express *KLKs* and their inhibitors, *SPINKs*. We found that in the absence of hormonal treatment, *KLK1* and *KLK7* were expressed at minimal levels in hEct1 and hEnd1 cells (Fig 7A). As expected, prostate-specific KLKs (*KLK2* and *KLK3*) were expressed only in the prostate cancer (LNCaP) cell line. hEct1 cells expressed *KLK4*, *KLK5*, and *KLK8* at high levels relative to other KLKs. hEnd1 cells also expressed *KLK4* and *KLK5*, but at lower levels, and showed a slightly higher level of *KLK8* compared to hEct1 cells. *SPINK5* and *SPINK6* were expressed in hEct1 cells. However, hEnd1 cells only expressed *SPINK5*, and not *SPINK6*. In addition, *KLK15* was expressed only in the LNCaP cells. Ishikawa (endometrial cancer) cells were used as a negative control.

hEct1 cells were chosen to assess whether E_2_ regulates the expression of *KLK*s and *SPINK*s based on their anatomical location, as they are the first cells semen comes in contact with after ejaculation. First, we identified whether hEct1 cells expressed *ESR1* in comparison to other cell types using RT-PCR. The transcript level of *ESR1* was approximately 15-fold higher in hEct1 cells compared to hEnd1 and LNCaP cell lines, and *ESR1* expression in Ishikawa cells remained below the detection threshold (Fig 7B). Protein levels of ESR1 were confirmed using immunoblotting (Fig 7C). The low level of β-ACTIN in the LNCaP cell line is likely due to the nature of LNCaP cells, which do not tightly adhere to the culture dish, and thus require fewer actin filaments.

After confirming the presence of ESR1 in hEct1 cells, hEct1 cells were treated with E_2_ in the presence or absence of ICI 182,780 (ICI; ESR antagonist) to inhibit the action of ESR1. E_2_ at a dose of 10 nM significantly increased the expressions of *KLK4*, *KLK5*, *KLK8*, *SPINK5*, and *SPINK6* (Fig 7D). In the presence of ICI, E_2_-induced expression of those genes was completely abolished (Fig 7D). Collectively, these results indicate that human cervical cells expressed distinct family members of *KLK*s compared to those in prostate cells, and the expression of KLKs and their inhibitors in ectocervical cells was regulated through E_2_/ESR1 action.

**Fig 8:**
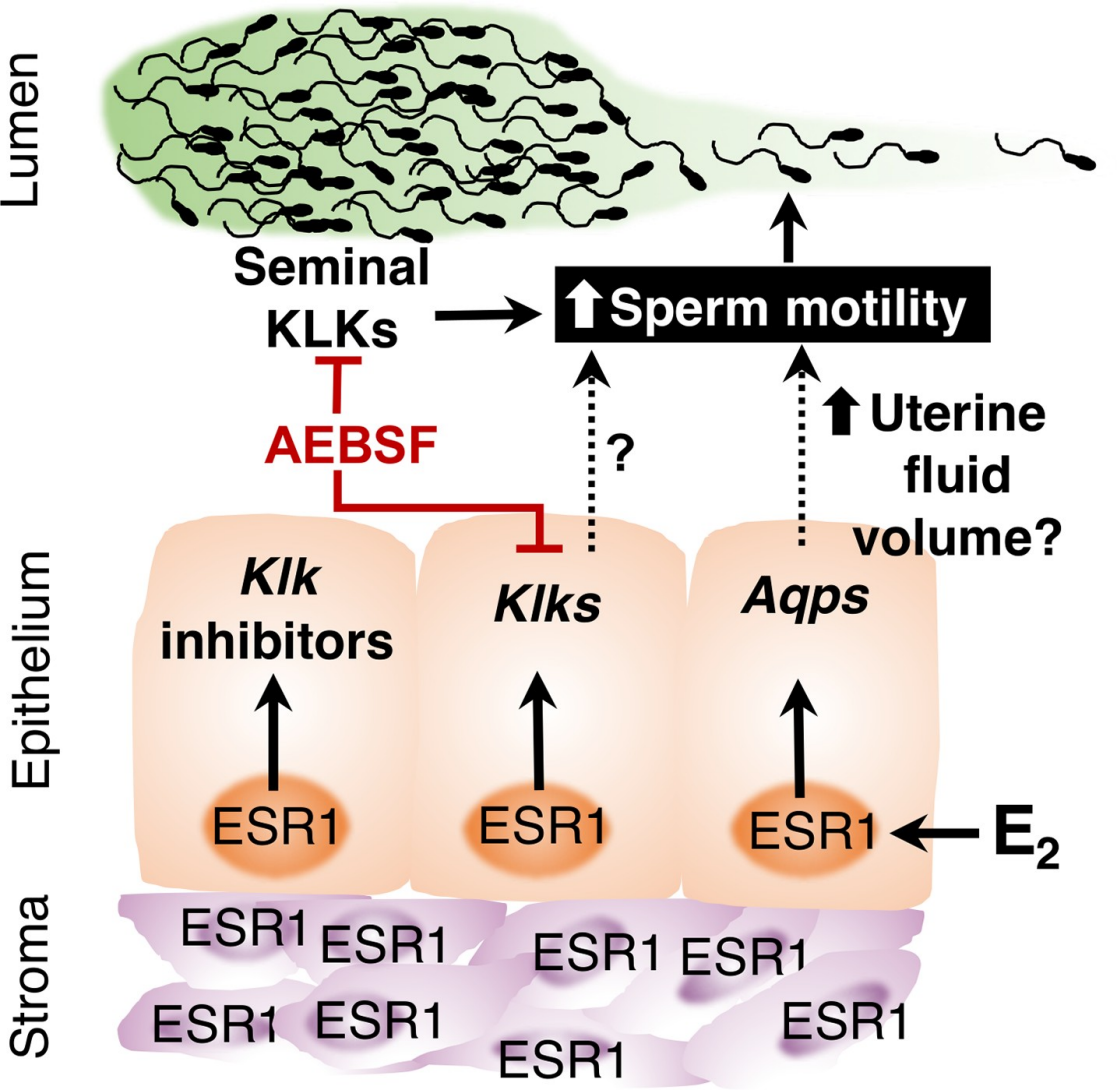
The proposed mechanisms of the semen liquefaction process in the female reproductive tract in the presence of normal E_2_/ESR1 signaling or serine protease inhibitor (AEBSF). Seminal KLKs activate liquefaction and increase sperm motility. The treatment of serine protease inhibitor in mouse uteri leads to an inhibition of seminal KLKs and uterine *Klks* (red lines). Increased uterine *Klks* could increase sperm motility by accelerating the liquefaction process. E_2_ signals through ESR1 in mouse uterine epithelial cells to increase the mRNA levels of *Klks* and KLK inhibitors. Water channel (aquaporins or *Aqps*) mRNA levels are also induced by E2 treatment, which may subsequently regulate the intraluminal uterine fluid through an increase in water transport. Increased uterine fluid flow could also promote sperm motility. Known information, supported by literature or the results of the present studies, is represented by solid lines. Proposed mechanisms are represented by dotted lines and question marks.

We propose a network of pathways to explain our observations of the process of semen liquefaction in the female reproductive tract (Fig 8). When semen, which contains KLKs, enters the uterus, it is exposed to uterine KLKs and KLK inhibitors that have been produced in response to E_2_ stimulation of ESR1. Normally, total KLK enzymatic activity overwhelms inhibitory action and produces liquefaction of the semen, which frees the sperm to swim into the oviduct. In addition, E_2_/ESR1 signaling may upregulate *Aqp* genes to increase fluidity of uterine contents and further support sperm movement into the oviduct. This proposed network should guide future exploration of the processes of liquefaction of semen and release of sperm to ascend to the site of fertilization.

## Discussion

In this study we evaluated the role of uterine epithelial ESR1 during semen liquefaction in the female reproductive tract to produce the following major findings: 1) E_2_ through ESR1 plays a crucial role in mammalian females by regulating semen coagulation and liquefaction and 2) *KLK*s and their inhibitors in human ectocervical cells are regulated by E_2_ in an ESR1-dependent manner.

### E_2_ signals in the female reproductive tract and semen liquefaction defect

Previous studies illustrated that female mice lacking ESR1 in the uterine and oviductal epithelial cells show a severely reduced number of sperm in the oviduct [20], however, the cause of this decrease was unclear. After ejaculation, sperm need to be dislodged from the seminal coagulum prior to traveling into the oviduct. In humans, semen from fertile men is completely liquefied after 20 to 30 minutes post-ejaculation *in vitro* [30]. It is known that liquefaction defects such as semen hyperviscosity is one of the causes of infertility in men [31], however, defective liquefaction caused by female conditions has never been reported. After ejaculation, semen is exposed to secretory proteins in the female reproductive tract, which possess a dynamic range of enzymatic activities including SPINKs [25]. Herein, we observed a liquefaction defect in *Wnt7a*^Cre/+^;*Esr1*^f/f^ uteri when these females mated with a male proven breeder, indicating that liquefaction can be modulated and disrupted by an exposure to a suboptimal environment in the female reproductive tract. Therefore, we proposed that these complex enzymatic activities in the female reproductive tract can disrupt post-ejaculated semen liquefaction. Our findings suggest that a lack of E_2_ signaling in the epithelial cells of the female reproductive tract causes faulty liquefaction and results in a fertility defect.

### Intrauterine fluid accumulation and *Aqp* gene expression

Due to a difference in uterine morphology between *Esr1*^f/f^ and *Wnt7a*^Cre/+^;*Esr1*^f/f^ females after mating, we investigated possible pathways that could lead to a lack of uterine ballooning or imbibition. One likely mechanism is E_2_-induced water transport channel activation. Astwood showed that luminal fluid volume is increased after 6 h of E_2_ treatment [21]. Later, Zhang *et al*. demonstrated that this E_2_-induced fluid accumulation is due to regulation through AQP5 and AQP8 by using *Aqp5*^*-/-*^ and *Aqp8*^*-/-*^ mouse models [22]. Analysis of our microarray data on the aquaporin gene family showed that E_2_ robustly induced the expression of *Aqp1*, *Aqp5*, and *Aqp8* 2 h after treatment in the uteri of ovariectomized control mice, and the effect declined 24 h after E_2_ treatment. However, these robust E_2_-induced *Aqp1*, *Aqp5*, and *Aqp8* transcripts were not observed in the absence of uterine epithelial ESR1. We did not observe differential expression of *Aqp* transcripts in the *Wnt7a*^Cre/+^;*Esr1*^f/f^ uteri after mating compared to controls. This is likely because the expression of *Aqps* had already declined to basal levels as the circulating level of E_2_ dropped after mating. These findings suggest that *Aqp1*, *Aqp5*, and *Aqp8* are upregulated by E_2_/ESR1 signaling. Zhang *et al*. demonstrated that *Aqp5*^−/−^;*Aqp8*^−/−^ mice are capable of having 80% of embryo implantation sites compared to controls, regardless of the significant decrease of intrauterine fluid accumulation [22]. However, Zhang *et al*. did not directly evaluate whether the reduction of uterine fluid disrupts sperm motility. Thus, the loss of fluid flow due to reduced uterine fluid volume could also result in the sperm motility defect observed in *Wnt7a*^Cre/+^;*Esr1*^f/f^ females.

### Expression of KLKs in the female reproductive tract is modulated by E_2_

KLKs are a family of serine proteases secreted from epithelial cells that play a central role in extracellular matrix remodeling, inflammation, and regulation of blood flow. There is limited knowledge regarding the proteolytic activities of seminal proteins after deposition in the female reproductive tract [32], therefore, it is likely that undetermined interactions between seminal proteins and secretions from the female reproductive tract may contribute to the liquefaction defect observed in *Wnt7a*^Cre/+^;*Esr1*^f/f^ female mice. In males, *KLK2* and *KLK3* transcripts are regulated by androgens; and androgen responsive elements (ARE) are present in the promoters of both KLK2 and 3 [33]. Conversely, KLK1 expression in mouse and rat uteri is influenced by E_2_ [17]; and an estrogen responsive element (ERE) is found in the *Klk1* promoter [34]. In humans, KLK levels in cervical and vaginal fluid are positively correlated with pregnancy and are possibly regulated by female steroid hormones [13].

As KLKs are secreted proteins, there is considerable overlap in the protein composition of fluids from the different parts of the female reproductive tract in both mice [35] and humans [25]. In mice, we demonstrated that mRNA levels of *Klk*s and their inhibitors are differentially expressed in the uterus and oviducts [20, 24]. Similar to mice, different KLKs are expressed in different cell types along the epithelial lining of the reproductive tract in women [12]. The secretory protein levels of KLKs correlate with the expression of each KLK member along the reproductive tract [25]. These findings suggest that the differential regulation of KLK expression in each region of the female reproductive tract also plays a crucial part in regulating local KLK activities.

Seminal KLKs inhabit a different environment from their site of secretion when they are deposited in the female reproductive tract. Our study showed that a lack of ESR1 in mouse uterine epithelial cells caused severely reduced expression of genes in the *Klk* family. However, our previous studies show that in the absence of epithelial ESR1 in the oviduct, expression of *Klks* is induced [20]. Therefore, these findings indicate that *Klk* expression in response to E_2_ is differentially regulated between the uterus and the oviducts. This novel finding suggests that E_2_-regulated *Klk* transcripts in the female reproductive tract are tightly regulated in a tissue-specific manner, and the induction of *Klks* through ESR1 in the uterus may be a mechanism for post-ejaculated semen liquefaction in the female reproductive tract. We speculate this differential regulation mechanism is used in part to locally regulate KLK activity.

### *Klk* mRNA expression and cell proliferation

E_2_ mediates the cell proliferation processes that are pivotal for cellular growth and differentiation. In both humans and mice, E_2_ increases the proliferation of cells in the female reproductive tract [36, 37]. Our previous work elucidates that normal uterine epithelial and stromal cell proliferation during early pregnancy is dependent on epithelial ESR1 [24]. To distinguish whether a loss of *Klk* production in the *Wnt7a*^Cre/+^;*Esr1*^f/f^ mice compared to *Esr1*^f/f^ mice was due to a difference in epithelial cell growth and proliferation, we assessed the proliferative index. We found the percentage of positive Ki67 luminal epithelial and stromal cells was comparable between *Esr1*^f/f^ and *Wnt7a*^Cre/+^;*Esr1*^f/f^ animals. However, in *Wnt7a*^Cre/+^;*Esr1*^f/f^ uteri, glandular epithelial cells had a significantly higher percentage of Ki67 positive cells compared to *Esr1*^f/f^ uteri. Thus to indirectly validate the gland formation, we evaluated the expression of *Foxa2*, a glandular epithelial cell marker [38], and observed a higher level of *Foxa2* expression in *Wnt7a*^Cre/+^;*Esr1*^f/f^ compared to *Esr1*^f/f^ uteri. We reasoned that elevated *Foxa2* expression as well as the glandular epithelial cell proliferation in the absence of epithelial ESR1 were due to a compensation for the lack of glandular products/secretions that normally provide positive feedback to stimulate uterine gland development. The increase in cell proliferation of glandular epithelium does not promote the expression of the *Klk*s in the mouse, and the change of *Klk* expression is not a secondary effect from cell proliferation due to a lack of ESR1.

### Other proteases and protease inhibitors in the female reproductive tract

Matsuda *et al*. demonstrated that 30 min of proteinase inhibitor treatment causes a solidification of human semen and results in a sperm motility defect [39]. In addition, the serine protease inhibitor SPINK3 is capable of inhibiting protease activity in the mouse uterus [40]. These findings indicate that the presence of protease inhibitors can disrupt semen liquefaction by suppressing protease activities from the semen. Our study demonstrated that the lack of ESR1 in epithelial cells of the female reproductive tract significantly increases the expression of the serine protease inhibitors *Serpinb7* and *Serpinh1*. However, the translational level of SERPINH1 protein was not different. Moreover, transgenic mice overexpressing *Serpinb7* showed normal fertility [41]. Thus, it is unlikely that the liquefaction defect in *Wnt7a*^Cre/+^;*Esr1*^f/f^ mice was due to excess protease inhibitors. In addition to serine proteases, other proteases like MMPs are also capable of degrading extracellular matrix proteins. Although MMP proteins do not possess the catalytic triads required for proteolytic function in KLKs (His57, Asp102, and Ser195) [42], neutrophils bearing proMMP9 can be attracted by seminal plasma and their activities are elevated post-coitus in the female uterus [43]. In our model, all *Mmp* transcripts and their inhibitors were expressed at the same level between *Esr1*^f/f^ and *Wnt7a*^Cre/+^;*Esr1*^f/f^ uteri. The only exception was a higher level of *Mmp2* in *Wnt7a*^Cre/+^;*Esr1*^f/f^ compared to *Esr1*^f/f^ uteri. MMP2 targets gelatin, collagen IV, V, VII, X and XI, fibronectin, laminin, elastin, and aggrecan [44]. Therefore, it is possible that the increased level of MMP2 transcripts was a response to the presence of an elevated collagen content (Fig 2A). Nevertheless, the elevated *Mmp2* level fails to correct the faulty semen liquefaction in the absence of uterine epithelial ESR1.

In addition to MMPs and SERPINs, the female reproductive tract also expresses several other proteases and protease inhibitors such as cathepsins, defensins, and SPLI, as part of their innate immune response to bacterial and viral infection [45]. Overall expression of these proteases and protease inhibitors was at comparable levels in *Esr1*^f/f^ and *Wnt7a*^Cre/+^;*Esr1*^f/f^ uteri. Minor changes in the transcripts for some of these genes did not alter our conclusion that a loss of E_2_ regulation in the uterine epithelial cells causes a disruption in *Klk* transcript levels, but not other proteases or protease inhibitors.

We investigated whether inhibition of multiple KLK activities in the uterus could mimic the phenotype of *Wnt7a*^Cre/+^;*Esr1*^f/f^ animals using AEBSF, an irreversible serine protease inhibitor that also affects tissue KLKs. We found that AEBSF treatment resulted in a drastic decrease in the number of sperm that reached the oviduct. However, the cleavage of SEMG1 in AEBSF treated animals was not significantly different from saline treated controls. It is possible that AEBSF could have unintended effects in the female reproductive tract. This could include inhibition of other serine proteases such as chymotrypsin and trypsin, which are present in semen and the uterus. This inhibitory effect could suppress the degradation of collagen and disrupt liquefaction. Moreover, post-mating uteri also contain the seminal KLKs. The suppression of KLK activities using AEBSF cannot rule out that liquefaction and semenogelin and collagen cleavage are catalyzed by seminal KLKs. Nevertheless, several proteases and protease inhibitors were present in both *Esr1*^f/f^ and *Wnt7a*^Cre/+^;*Esr1*^f/f^ uteri, and the solidified semen was only observed in the condition where uterine *Klks* were minimally expressed.

### Sperm entrapment and a lack of SEMG cleavage

Histological analysis demonstrated a semen liquefaction defect of entrapped sperm within the semen coagulum in the uterus in the absence of epithelial ESR1. The denser semen resulted in a prolonged liquefaction time in *Wnt7a*^Cre/+^;*Esr1*^f/f^ animals. The majority of sperm entrapped in the solidified semen were immotile as they were unable to dislodge from the seminal coagulum and the higher sperm density in *Wnt7a*^Cre/+^;*Esr1*^f/f^ uteri was attributed to a reduced fluid volume in the uterus. There was no statistically significant difference between total sperm number in *Wnt7a*^Cre/+^;*Esr1*^f/f^ and *Esr1*^f/f^ uteri. We further identified that gel-forming proteins in the seminal plasma, SEMG1 and collagen, were not degraded in *Wnt7a*^Cre/+^;*Esr1*^f/f^ uteri. Collectively, these results indicate that ejaculated semen requires additional regulation in the female reproductive tract to lyse gel-forming proteins and allow effective liquefaction.

### Human ectocervical cells express *KLKs* and are regulated through ESR1

In women, post-ejaculated semen is deposited at the anterior wall of vagina adjacent to the ectocervix. To explore whether *KLK*s are also expressed and regulated by E_2_/ESR1 signaling in human cervical cells, we determined the expression pattern of all KLK family members using immortalized hEct1 and hEnd1 cells. The results indicated that KLKs were expressed in the hEct1 cells and that *KLK4*, *KLK5*, and *KLK8* were the most abundant serine proteases. This finding is relevant to reproductive medicine as it was previously shown that KLK4, KLK5, and KLK8 are able to cleave fibronectin [46], another important protein that contributes to semen coagulation [47]. KLK5 is also responsible for digestion of collagen and modification of mucin to facilitate sperm transport [13, 25]. In addition, Pro-KLK2, 3, 7, 8, and 14 in the semen are activated by KLK5 [48], which is self-activated over time [9].

More importantly, this study indicates that KLK inhibitors *SPINK5* and *SPINK6* were highly expressed in the hEct1 cell line. E_2_ increases *KLK* and *SPINK* expression through an ESR1-dependent pathway. SPINK5 specifically inhibits KLK5, KLK7, and KLK14 activity, and mutations in *SPINK5* cause Netherton Syndrome, a severe skin disorder [49, 50]. The function of SPINK6 is less well-established [51]. Although human seminal fluid contains KLK2, 3, 4, 5, 8, 11, 12, 14, and 15 [52], the function of SPINKs in semen liquefaction is unclear. Inhibition of KLK5 by SPINK5 and 6 can prevent the activation of other KLKs in the semen. Ultimately, inhibition of KLK5 could potentially lead to two problems: 1) a failure to activate KLK2 and 3, preventing the degradation of SEMG and causing faulty semen liquefaction and sperm liberation, and 2) a defective cervical mucin remodeling, as KLK5 and 12 are responsible for cleavage of mucin 4 and 5B, the main cervical mucus proteins [25, 53] to guide the sperm to go through the cervix. These findings strongly suggest that human KLKs and KLK inhibitors from the female reproductive tract are indispensable for normal semen liquefaction and sperm transport.

### Conclusion

This study demonstrated a novel interaction between semen and the microenvironment in the female reproductive tract. It uncovered a post-ejaculated semen liquefaction process that occurs inside the female reproductive tract, a topic that has not yet been explored. The conclusion of the experiments proposed that abnormal E_2_ signaling in the female reproductive tract leads to a semen liquefaction defect associated with defective SEMG cleavage and sperm transport and may be a possible cause for some cases of infertility. We proposed the explanation that the semen liquefaction defect could be caused by diminished KLK activity in female mice, however, this hypothesis should first be tested in multiple *Klk* knockout mouse models. This discovery regarding post-ejaculated liquefaction is crucial for advancing the field of reproductive medicine and can lead to potential diagnostic tools for unexplained infertility cases and to developing a novel contraception technology to entrap sperm.

## Materials and methods

### Ethics statement

Animals were handled according to Washington State University (WSU) Animal Care and Use Committee guidelines and in compliance with WSU-approved animal protocols #4702 and 4735.

### Animals and experimental procedures

Adult female mice (7 to 12 weeks old) with a selective deletion of ESR1 in the epithelial cells of the female reproductive tract (*Wnt7a*^Cre/+^;*Esr1*^f/f^) [19] and their control littermates (*Esr1*^f/f^) were used in the experiments. The genotyping of the animals was carried out as previously described [19]. The deletion of ESR1 was confirmed using IHC analysis (details described below). Female mice were singly housed and bred overnight with a wild-type (WT) C57B6/J male. If a copulatory plug was observed the next morning at 8 a.m., the female was designated as 0.5 days post coitus (dpc). For the microarray analysis, the animals were ovariectomized, randomly divided into groups, and treated with the vehicle control (sesame oil) or 17β-estradiol (E_2_ at a dose of 0.25 μg/mouse) for 2 and 24 h as described previously [24]. Microarray data in this publication were deposited in NCBI's Gene Expression Omnibus and are accessible through GEO Series accession number GSE23072 (WT samples) and GSE53812 (*Wnt7a*^Cre/+^;*Esr1*^f/f^ samples). The differentially expressed genes and statistical analysis for the microarray dataset were analyzed using Partek Genomics Suite (version 6.6*Beta* 6.11.1115, St. Louis, MO) as described previously [24].

At the time of tissue collection, animals were euthanized using CO_2_ asphyxiation with cervical dislocation. Uterine diameters were measured using a ruler before tissue dissection. Semen was collected from the uteri and liquefaction time was determined by recording the time used to fill a 25 μL capillary tube using an adapted method as previously reported [54]. One uterine horn from each animal was snap frozen and stored at -80°C for RNA and protein extraction. Contralateral horns were fixed in 10% phosphate-buffered formalin for histological analysis.

### Histological procedures, IHC staining, and quantitative analysis of Ki67 expression

Formalin-fixed uterine tissues were dehydrated and embedded in paraffin. The tissues were cross-sectioned at 5 microns and stained with hematoxylin and eosin (H&E) using a standard histological protocol. For IHC staining, paraffin sections were rehydrated and the antigen was retrieved using citrate buffer in the decloaking chamber (BioCare Medical, Concord, CA). Endogenous peroxidase activity was blocked using 3% H_2_O_2_. The uterine sections were blocked with 10% normal horse serum (NHS) diluted in automation buffer (50 mM Tris, 20 mM NaCl, 0.05% Tween-20) for 1 h at room temperature, then incubated with the primary antibodies against ESR1 (1:200, # MA5-13191, ThermoFisher Scientific, Carlsbad, CA) or Ki67 (1:200, #550609, BD Pharminogen, San Jose, CA) in 10% NHS for 1 h at room temperature. Mouse IgG was used in place of primary antibodies for a negative control. The secondary antibody (1:1000 biotinylated horse anti-mouse, Vector Laboratories, Burlingame, CA) was applied to the sections for 30 min at room temperature. Vectastain RTU Elite and ImmPact kits (Vector Laboratories) were used according to the manufacturer’s protocols to detect the positive signals. Tissues were counterstained with hematoxylin, dehydrated, and coverslipped with Permount. The presence of collagen in the uterine content was determined using Masson’s Trichrome staining kit. Blue staining indicates the presence of collagen and red indicates cytoplasm. Quantification of Ki67 IHC was determined using ImageJ version 2.0.0-rc-43/1.51e (imagej.nih.gov) with Cell Counter Tool Plugins as previously described [24]. Three images from each animal were captured using the Leica Application Suite (Leica Microsystems Inc., Buffalo Grove, IL). The number of Ki67 positive cells was counted and calculated as the percentage of positive cells per each cell type (luminal epithelial, glandular epithelial, and stromal cells) in each image.

### Semen collection, sperm number, and sperm motility analysis

Semen was collected from the uteri of *Esr1*^f/f^ and *Wnt7a*^Cre/+^;*Esr1*^f/f^ females approximately 8 h after mating. The female reproductive tract including the vagina was collected. The area between the uterus and cervix was cut open into a 1.5 mL microcentrifuge tube in order to collect the semen. The uterine horns were squeezed using toothless forceps from the ovarian end to the vaginal end to extract semen from the uterine lumen. The tubes were spun down briefly and the uterine fluid volume was determined. The semen was then diluted 1:200 in diH_2_O and the number of sperm was counted using a hemocytometer. The total sperm number was calculated and normalized to the total fluid volume collected. Simultaneously, another 5 μL of fresh semen was dropped onto a glass slide and covered with a coverslip. Bright field video images of sperm motility were recorded with the 100x objective lens (4–8 different microscopic fields/sample) using a DMi8 Leica Microscope (Leica Microsystems). The sperm motility within each microscopic field was sorted into four categories: immotile, non-progressive, slow progressive, and rapid progressive, and the percentage of sperm in each category was calculated. The total number of sperm evaluated was 339 for *Esr1*^f/f^ and 414 for *Wnt7a*^Cre/+^;*Esr1*^f/f^ groups (n = 3 females/genotype). The time between animal euthanasia and sperm video recording was within 5 min to preserve the maximal sperm viability.

### Sperm density

The sperm density was determined from H&E stained tissues collected from 0.5 dpc uteri and observed with the 20x objective lens. The genotype of the animals was blinded from the observer. The number of sperm within the uterine lumen was counted manually using winDRP software (v1.6.4) [55], the total area was measured and used to calculate the sperm density per mm^2^. Data were collected from 7 *Esr1*^f/f^ and 10 *Wnt7a*^Cre/+^;*Esr1*^f/f^ animals (1 image per animal).

### Intraluminal treatment of AEBSF

Adult female C57BL6/J mice (7–12 weeks old) were used. The estrus stage of the estrous cycle was determined using a vaginal smear. At 5 p.m. on the determined day of estrus, females were administered 15 μL of saline pH 5.5 or 15 μL of AEBSF (4-(2-Aminoethyl)benzenesulfonyl fluoride hydrochloride, Amresco, Dallas, TX) at a dose of 300 μg/mouse through transcervical treatment using a Non Surgical Embryo Transfer (NSET) device [56]. The 300 μg dose of AEBSF was selected based on the demonstration of Sun *et al*. that a single intraluminal injection effectively inhibits embryo-uterine implantation [57]. The females were immediately housed with a proven male breeder overnight. The next morning, presence of a copulatory plug was designated as 0.5 dpc. The animals were euthanized at 8 a.m. and the uteri were collected as described above. The oviducts were flushed with 100 μL of diH_2_O onto the glass slide. The total number of sperm in the oviduct was counted manually under a brightfield microscope with the 20x objective lens.

### Gene expression analysis using RT-PCR

RNA was extracted from the uteri or human cell lines using RiboZol ME Reagent (Amresco, Solon, OH) according to the manufacturer’s protocol. A total of 1 μg RNA was used as a template for the reverse transcriptase reaction according to the manufacturer’s protocol (qScript cDNA SuperMix, QuantaBio, Beverly, MA). The cDNA products were diluted at 1:10 in nuclease-free H_2_O. Diluted cDNA (1 μL) was used as a template for the PCR reaction (PerfeCTa SYBR Green FastMix, QuantaBio) according to the manufacturer’s protocol. PCR reactions were run and raw data were recorded in a 7500 Fast Real-Time PCR System (Applied Biosystems, ThermoFisher). Expression values in uterine samples were calculated as fold change normalized to ribosomal protein L7 (*Rpl7*) expression, relative to the vehicle or *Esr1*^f/f^. Expression of human *KLKs*, *SPINKs*, and *ESR1* was calculated relative to *RPL5* expression. The primer sequences used in the experiments are listed in S2 Table.

### Immunoblotting

Protein was extracted from uterine tissues using T-PER tissue protein extraction reagent (ThermoFisher) with Halt protease and phosphatase inhibitors (ThermoFisher). The protein concentration was determined using a BCA protein assay (ThermoFisher). A total of 20 μg protein was loaded into each lane of a 10% acrylamide gel. The protein gels were transferred to a nitrocellulose membrane using Trans-Blot Turbo (Bio-Rad Laboratories Inc., Hercules, CA). To visualize the equal protein loading and transfer, the membranes were incubated with 0.1% Ponceau S in 1% acetic acid. Membranes were then blocked with 5% non-fat dry milk in Tris-buffered saline with 0.1% tween-20 (TBST) for 1 h at room temperature. The membranes were then incubated with primary antibodies (1:1000 of KLK1B5 #MBS175442, MyBioSource, San Diego, CA; 1:1000 of SERPINH1 (or HSP47), # MAB9166, R&D Systems, Minneapolis, MN; 1:2000 of β-ACTIN #SC-47778, Santa Cruz Biotechnology, Inc., Dallas, TX, 1:1000 of SEMG1 #ab139405, Abcam, Cambridge, MA) in 5% milk TBST at 4°C overnight. The secondary antibodies were incubated for 1 h at room temperature with a dilution of 1:5000 in 5% milk TBST. The blotting was detected using Amersham ECL Select (GE Healthcare Bio-Sciences, Pittsburgh, PA). The chemiluminescent signals were detected and images were captured using ChemiDoc MP System (Bio-Rad).

### Human cell cultures

Immortalized human ectocervical (hEct1) and endocervical (hEnd1) cells were purchased from ATCC (Manassas, VA). Human endometrial cancer (Ishikawa) cells derived from the 3H12 clone [58] and human prostate cancer (LNCaP) cells (ATCC) were used as controls for the experiments. Immortalized hEct1 and hEnd1 cells were cultured in keratinocyteserum-free medium (ThermoFisher) with 0.1 ng/mL human recombinant epidermal growth factor, 0.05 mg/mL bovine pituitary extract, and 0.4 mM calcium chloride (complete KSFM) at 37°C with 5% CO_2_. Ishikawa and LNCaP cells were cultured in RMPI medium (Hyclone, South Logan, UT) supplemented with 10% fetal bovine serum (Gemini Bio-Products Inc., Woodland, CA), 100 U/mL penicillin, and 100 μg/mL streptomycin. Cells were cultured to 80–90% confluence, washed with phosphate buffered saline (PBS), and harvested using 0.25% trypsin-EDTA (Sigma, St. Louis, MO). RNA was extracted using RiboZol ME as described above.

For the analysis of endogenous gene expression in hEct1 cells in response to E_2_, the cells were plated at a density of 1.33x10^6^ cells/mL in a 6-well plate containing complete KSFM media. Cells were grown to 80–90% confluence, then treated with vehicle (0.1% ethanol) or E_2_ (1 and 10 nM) in the presence or absence of ICI 182,780 (ICI; 1 μM) for another 24 h. Cells were then harvested and RNA was extracted using RiboZol ME as indicated above.

### Statistical analysis

Data are represented as mean±SEM and analyzed using GraphPad Prism version 6.0 for Mac OS X. Data were evaluated for statistically significant differences (*p*<0.05) using an unpaired student *t-*test, unless otherwise indicated.

## Supporting information

S1 Video Representative video recording of the semen freshly collected from control uteri approximately 8 h after mating at 1000x magnification with a speed of 12 frames/second (n = 3 females). (AVI)

S2 Video Representative video recording of the semen freshly collected from *Wnt7a*^Cre/+^;*Esr1*^f/f^ uteri approximately 8 h after mating at 1000x magnification with a speed of 12 frames/second (n = 3 females). (AVI)

S1 Table Microarray analysis showing all the transcript levels^π^ of aquaporins detected in the uterus of *Esr1*^f/f^ and *Wnt7a*^Cre/+^;*Esr1*^f/f^ female 2 h and 24 h after E_2_ treatment. π All the transcripts detected in the gene family in the uteri were listed in the table regardless of the fold changes. Transcript levels indicated by the raw signal intensities; cut off values were ≤ 100 in both groups.
(DOCX)

S2 Table List of primer sequences^π^ used in this study. π *m* and *h* prefixes refer to mouse and human primers, respectively.
(DOCX)
