# Supplementary material for: Crucial role of estrogen for the mammalian female in regulating semen coagulation and liquefaction in vivo
Source: PLoS Genet. 2017 Apr 17;13(4):e1006743. doi: 10.1371/journal.pgen.1006743 (PMC5411094; doi:10.1371/journal.pgen.1006743)
Supplement: S1 Table — π All the transcripts detected in the gene family in the uteri were listed in the table regardless of the fold changes. Transcript levels indicated by the raw signal intensities; cut off values were ≤ 100 in both groups. (DOCX) [file pgen.1006743.s003.docx]

| **Accession #** | **Primary Sequence Name** | ***Esr1*^f/f^** | | ***Wnt7a*^Cre/+^;*Esr1*^f/f^** | | **Fold Change** |
| --- | --- | --- | --- | --- | --- | --- |
|  |  | **Signal intensity** | ***p*-value** | **Signal intensity** | ***p*-value** |  |
| **Aquaporins (*Aqp*) after E_2_ 2h** | | | | | | |
| NM_007472 | *Aquaporin 1* | 102,092.3 | 2.90E-12 | 73,781.2 | 9.56E-18 | -1.4 |
| NM_009699 | *Aquaporin 2* | 456.7 | 1.30E-16 | 470.2 | 2.72E-26 | 1.0 |
| NM_016689 | *Aquaporin 3* | 947.8 | 2.43E-37 | 1062.7 | 2.03E-37 | 1.1 |
| NM_009701 | *Aquaporin 5* | 87,093.6 | 5.17E-18 | 25,381.8 | 3.00E-06 | -3.4 |
| NM_007474 | *Aquaporin 8* | 11,666.1 | 1.74E-17 | 577.2 | 2.69E-38 | -20.2 |
| NM_175105 | *Aquaporin 11* | 784.0 | 9.77E-33 | 1,799.9 | 9.60E-13 | 2.3 |
| **Aquaporins (*Aqp*) after E_2_ 24h** | | | | | | |
| NM_007472 | *Aquaporin 1* | 55,203.1 | 0.0E+00 | 42,337.3 | 0.0E+00 | -1.9 |
| NM_009699 | *Aquaporin 2* | 325.5 | 0.0E+00 | 373.7 | 0.0E+00 | 1.0 |
| NM_016689 | *Aquaporin 3* | 798.7 | 9.89E-38 | 880.6 | 5.65E-23 | 9.2 |
| NM_009701 | *Aquaporin 5* | 14,085.7 | 0.0E+00 | 30,251.7 | 0.0E+00 | 1.9 |
| NM_007474 | *Aquaporin 8* | 1,744.6 | 9.53E-14 | 311.9 | 1.26E-44 | 1.2 |
| NM_175105 | *Aquaporin 11* | 1,731.9 | 8.20E-13 | 1,863.7 | 0.0E+00 | -1.1 |
