# Supplementary material for: Crucial role of estrogen for the mammalian female in regulating semen coagulation and liquefaction in vivo
Source: PLoS Genet. 2017 Apr 17;13(4):e1006743. doi: 10.1371/journal.pgen.1006743 (PMC5411094; doi:10.1371/journal.pgen.1006743)
Supplement: S2 Table — π m and h prefixes refer to mouse and human primers, respectively. (DOCX) [file pgen.1006743.s004.docx]

| **Gene names** | **Primer sequences** |
| --- | --- |
| *mAqp1* | F: ACCTGCTGGCGATTGACTAC  R: TGGTTTGAGAAGTTGCGGGT |
| *mAqp5* | F: ACCAGATCTCTCTGCTCCGA  R: GTTGTTGCTGAGCGCATTGA |
| *mAqp8* | F: ACTTGTGGGCTCCGCTCTCT  R: CTGGACGATGGCAAAGGCT |
| *mAqp11* | F: GCTTGCTCCTTCTGTAGGTGT  R: GCTTGACTGTCCTGGGACTT |
| *mKlk1* | F: ACACCCGTCAAATATGAATACCCA  R: CACCCGCACAAGTGTCTTTG |
| *mKlk1b5* | F: TGCAGGAGATATGGATGGAGGCAA  R: ACCGTGGAGAACACCATCACAGAT |
| *mMmp2* | F: CAAGTTCCCCGGCGATGTC  R: TTCTGGTCAAGGTCACCTGTC |
| *mMmp9* | F: GCGTGTACGGACCCGAAG  R: AGGGATACCCGTCTCCGTG |
| *mRpl7* | F: AGCTGGCCTTTGTCATCAGAA  R: GACGAAGGAGCTGCAGAACCT |
| *mSerpina1d* | F: TTAAAGGCAAATGGAAACAGC  R: CTTCACTGTGGTGGACTCGT |
| *mSerpinh1* | F: AACTGTCACTTGCCTGGGTT  R: GGACCTGTGAGGGTTTACCA |
| *mTimp1* | F: GCAGATATCCGGTACGCCTACA  R: TGCGGTTCTGGGACTTGTG |
| *hKLK1* | F: ACCTGGAAGGTGGCAAAGAC  R: AGAAGGCTTATTGGGGGTGC |
| *hKLK2* | F: TGGCTGTGTACAGTCATGGA  R: ATATTGTAGAGCGGGTGTGGG |
| *hKLK3* | F: CCCACTGCATCAGGAACAAAA  R: CTCATATCGTAGAGCGGGTGT |
| *hKLK4* | F: TGGTAGCTGCAGCCAAATCA  R: GTTCTGGAAACAGTGTGCGG |
| *hKLK5* | F: AGGGAGCAGTGGGTGGTTAT  R: ACCCTCACCAGGTCTCACTT |
| *hKLK6* | F: CGGACAAAGCCCGATTGTTC  R: TCTTGAGTCGGGGGAAGGAA |
| *hKLK7* | F: CTGCGTCAGGGTTCCAGATA  R: ACACAGCTGGGCTCCAAAAT |
| *hKLK8* | F: AGACGTGGATGTTCCTGCTC  R: GCCACTGTGGGTTCAAATGG |
| *hKLK9* | F: GGACACATCTCGGACAGCAT  R: GTGGCATACGCTGGTGTAGA |
| *hKLK10* | F: GCACTGGGAGAAGCCTGTAT  R: CAGAGTTGCGCCATCAGCAG |
| *hKLK11* | F: AACCCAGCCTACCTGCTGTA  R: GCACTCGAACCCCTTGATGA |
| *hKLK12* | F: CCACCTGCCATGGTGTGTAT  R: CAAGAGTGGAGTTGCAAATATAGGT |
| *hKLK13* | F: ACTGCCGCACACTGTCTAAA  R: TCATGGTCGTGGTTCAGGTG |
| *hKLK14* | F: CCTCGTGTCTTGGGGAATGG  R: GGAGAAAACGCCTGAAGCAA |
| *hKLK15* | F: GCCCAGGATGGTGACAAGTT  R: AGGGAAGCGCCACAGTTAAA |
| *hSPINK5* | F: TGGAAAACTGTTCTGTCCCCA  R: AAATGCCTGGCCCTCTTCTG |
| *hSPINK6* | F: GCCGGGAGGATGTATTGGTT  R: TTTAATGGGGCCTGCCAACA |
| *hRPL5* | F: GAGGTGGAACCGTCCCAAAA  R: GCTGGGTTTAGCTCTCAGCA |
| *hESR1isoform1(variant1)* | F: TTCGTCCTGGGACTGCACTTG  R: AGCACAGCCCGAGGTTAGAG |
| *hESR1isoform1(variant2)* | F: CTGGATCCGTCTTTCGCGTT  R: TTGTCGTCGCTGCTGGATAG |
| *hESR1isoform1and2* | F: TCTCTCGGCCCTTGACTTCTA  R: TAGGGCCATCCCAGATGCTT |
| *hESR1isoform3* | F: GGGAATGATGAAAGGGATACGA  R: AAAGGTTGGCAGCTCTCATGT |
| *hESR1isoform4* | F: GCTGGCTACATCATCTCGGT  R: GGCCTTATGACCAGAGGCTT |
